# SLAMF7 regulates the inflammatory response in macrophages during polymicrobial sepsis

**DOI:** 10.1172/JCI150224

**Published:** 2023-02-07

**Authors:** Yongjian Wu, Qiaohua Wang, Miao Li, Juanfeng Lao, Huishu Tang, Siqi Ming, Minhao Wu, Sitang Gong, Linhai Li, Lei Liu, Xi Huang

**Affiliations:** 1 Center for Infection and Immunity and Guangdong Provincial Key Laboratory of Biomedical Imaging, The Fifth Affiliated Hospital of Sun Yat-sen University, Zhuhai, Guangdong Province, China.; 2 Scientific Research Center, The Seventh Affiliated Hospital, Sun Yat-sen University, Shenzhen, Guangdong Province, China.; 3 National Clinical Research Center for Infectious Disease, Shenzhen Third People’s Hospital, The Second Affiliated Hospital of the Southern University of Science and Technology, Shenzhen, Guangdong Province, China.; 4 Department of Gastroenterology, Guangzhou Women and Children’s Medical Center, Guangzhou Institute of Pediatrics, Guangzhou Medical University, Guangzhou, Guangdong Province, China.; 5 The Sixth Affiliated Hospital of Guangzhou Medical University, Qingyuan People’s Hospital, Qingyuan, Guangdong Province, China.

**Keywords:** Infectious disease, Inflammation, Bacterial infections, Macrophages

## Abstract

Uncontrolled inflammation occurred in sepsis results in multiple organ injuries and shock, which contributes to the death of patients with sepsis. However, the regulatory mechanisms that restrict excessive inflammation are still elusive. Here, we identified an Ig-like receptor called signaling lymphocyte activation molecular family 7 (SLAMF7) as a key suppressor of inflammation during sepsis. We found that the expression of SLAMF7 on monocytes/macrophages was significantly elevated in patients with sepsis and in septic mice. SLAMF7 attenuated TLR-dependent MAPK and NF-κB signaling activation in macrophages by cooperating with Src homology 2–containing inositol-5′‑phosphatase 1 (SHIP1). Furthermore, SLAMF7 interacted with SHIP1 and TNF receptor–associated factor 6 (TRAF6) to inhibit K63 ubiquitination of TRAF6. In addition, we found that tyrosine phosphorylation sites within the intracellular domain of SLAMF7 and the phosphatase domain of SHIP1 were indispensable for the interaction between SLAMF7, SHIP1, and TRAF6 and SLAMF7-mediated modulation of cytokine production. Finally, we demonstrated that SLAMF7 protected against lethal sepsis and endotoxemia by downregulating macrophage proinflammatory cytokines and suppressing inflammation-induced organ damage. Taken together, our findings reveal a negative regulatory role of SLAMF7 in polymicrobial sepsis, thus providing sights into the treatment of sepsis.

## Graphical abstract

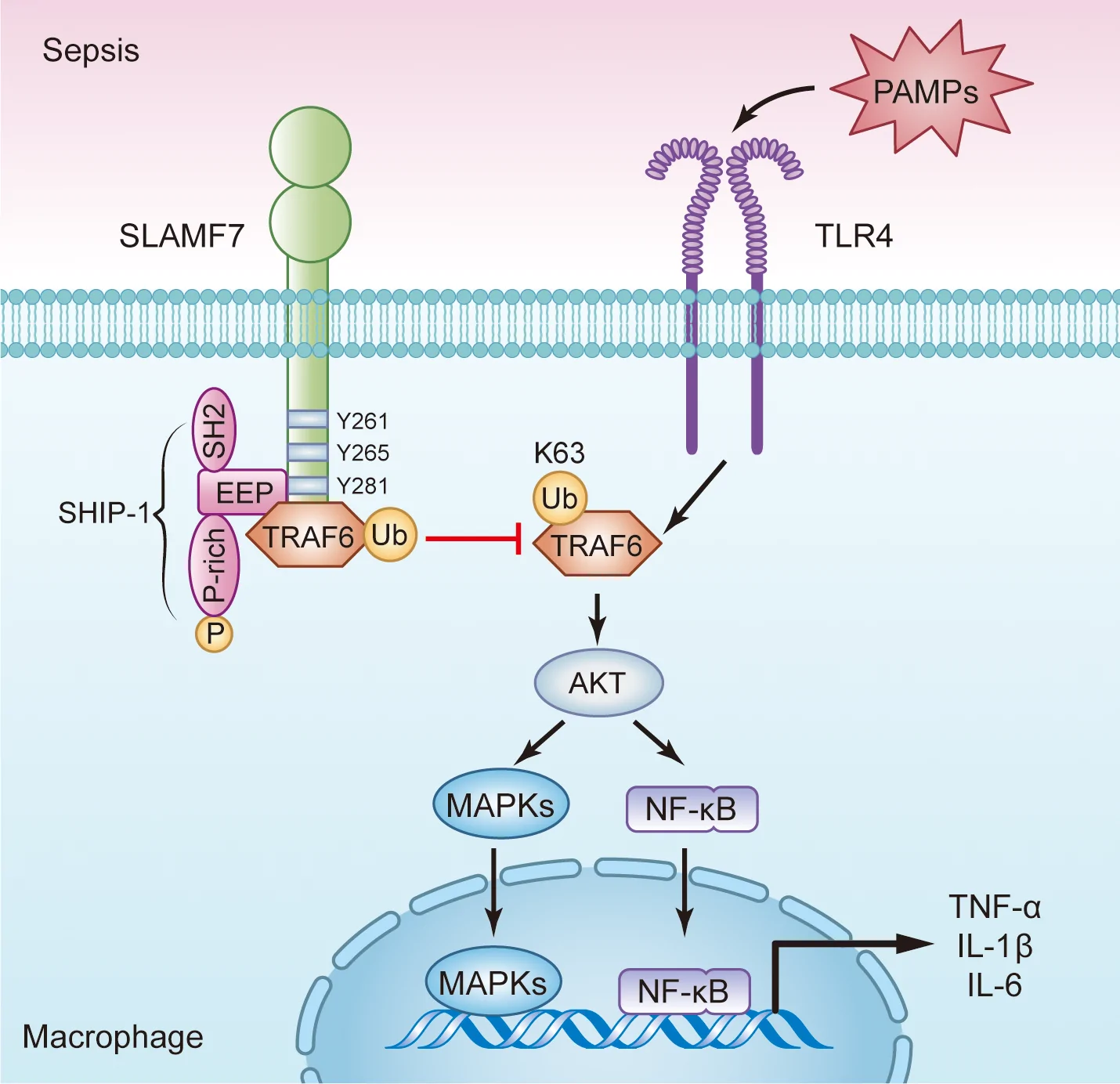

## Introduction

Sepsis is one of the leading causes of in-hospital death, but the pathological mechanisms underlying this disease have yet to be uncovered (1). Defining the clinical criteria for the sepsis remains challenging, as does determining the timing of its onset (2). Currently, sepsis is defined as a life-threatening organ dysfunction due to a dysregulated host immune response to infection (3, 4). Clearance of pathogens leading to sepsis relies heavily on the activation of the innate immune response by pattern recognition receptors (PRRs) that recognize microbial pathogens, especially endotoxin and the Gram-negative bacilli *E*. *coli* and *Pseudomonas aeruginosa* (5). TLRs play a critical role in the response to both exogenous pathogens and endogenous ligands. Inflammatory mediators including cytokines, chemokines, and growth factors released by immune cells upon TLR activation are beneficial in the eradication of pathogens. However, excessive inflammatory responses can lead to organ failure and even death in patients with sepsis. In the past 4 decades, therapies targeting TLRs and the inflammatory response have been widely explored in sepsis (6). There have been more than 100 phase II and phase III clinical trials attempting to modify the systemic inflammatory response by selectively or nonselectively targeting its endogenous mediator molecules (6). For example, since TLR4 induces excessive inflammation in sepsis (7), antagonists targeting TLR4 have been used in a phase II clinical trial that aims to decrease the mortality of patients with sepsis (8). However, the effects of most therapeutic targets have not been satisfactory in clinical trials so far (9). Therefore, further explorations of inflammation-modulatory mechanisms underlying sepsis are urgently needed to develop more efficient therapies.

During the process of innate immune response, a variety of negative effector molecules such as VEGFR-3 (10) and MHC class II (MHC-II) (11) are induced and act as feedback loops to dampen inflammation induced by sepsis. Signaling lymphocytic activation molecule family (SLAMF) receptors, a subgroup of CD2 superfamily Ig-like receptors, have been demonstrated to modulate immune responses in health and diseases (12, 13). Among them, SLAMF7 (also named CS1, CRACC, and CD319) plays a critical role in the function regulation of immune cells, especially NK cells (14–16). Like other SLAMF members, SLAMF7 functions as a self-ligand receptor. SLAMF7 contains immunoreceptor tyrosine-based switch motifs (ITSMs) in the cytoplasmic domain, which are phosphorylated upon activation and recruit SH2 domain–containing molecules to transmit downstream signals (17, 18). The most common adaptors for SLAMF receptors are Ewing’s sarcoma-associated transcript 2 (EAT-2) and SLAM-associated adaptor protein (SAP). Cytoplasmic ITSM of SLAMF7 only binds to EAT-2, but not SAP, in the presence of EAT-2 to activate NK cells (17). Studies have focused on the functions of SLAMF7 as an important immune checkpoint in multiple myeloma (MM) immunotherapy (19, 20). The mechanism of elotuzumab (a humanized monoclonal antibody against SLAMF7) is thought to enhance the activation and natural killing activities of NK cells (18, 21). Furthermore, SLAMF7 interacts with integrin Mac-1 in macrophages and utilizes signals involving responses for tumor cell phagocytosis (19). Interestingly, SLAMF7 also acts as an inhibitory receptor in the absence of EAT-2 via the recruitment of a number of inhibitory phosphatases (SHP1, SHP2, SHIP1, and Csk) (18). SLAMF7 has been proven to inhibit TNF and IL-12p70 expression in human monocytes (16), although the specific mechanisms remain undefined. Moreover, SLAMF7 exhibits a negative regulatory effect on inflammation in some infectious diseases (22, 23). For instance, increased SLAMF7 expression in macrophages alleviates corneal inflammation by promoting M2 polarization (23). In addition, SLAMF7 downregulates IFN-α–mediated CXCL10 production in chronic HIV infection (22). Nevertheless, the underlying mechanisms of SLAMF7 in inflammatory response regulation are still unclear.

In this study, we observed upregulated expression of SLAMF7 in sepsis and demonstrated that SLAMF7 negatively regulated sepsis-induced inflammation. SLAMF7 expression was elevated in both monocytes from patients with sepsis and macrophages from septic mice. Meanwhile, SLAMF7 expression was induced by TLR receptors and was tightly related to the progress of sepsis. Further explorations showed that SLAMF7 interacted with SHIP1 to inhibit TRAF6 autoubiquitination, thus reducing TLR-triggered inflammatory responses via the inhibition of NF-κB and MAPK activation in macrophages. In vivo experiments revealed that activation of SLAMF7 by recombinant protein reduced mortality and protected mice from an excessive inflammatory response and organ damage. Consistently, SLAMF7 KO in mice reduced their survival rate and aggravated sepsis by amplifying inflammation. In summary, our results uncover the negative role of SLAMF7 in sepsis and inflammation, which may provide therapeutic strategies for sepsis treatment.

## Results

### SLAMF7 expression is strongly related to sepsis.

**Figure 1:**
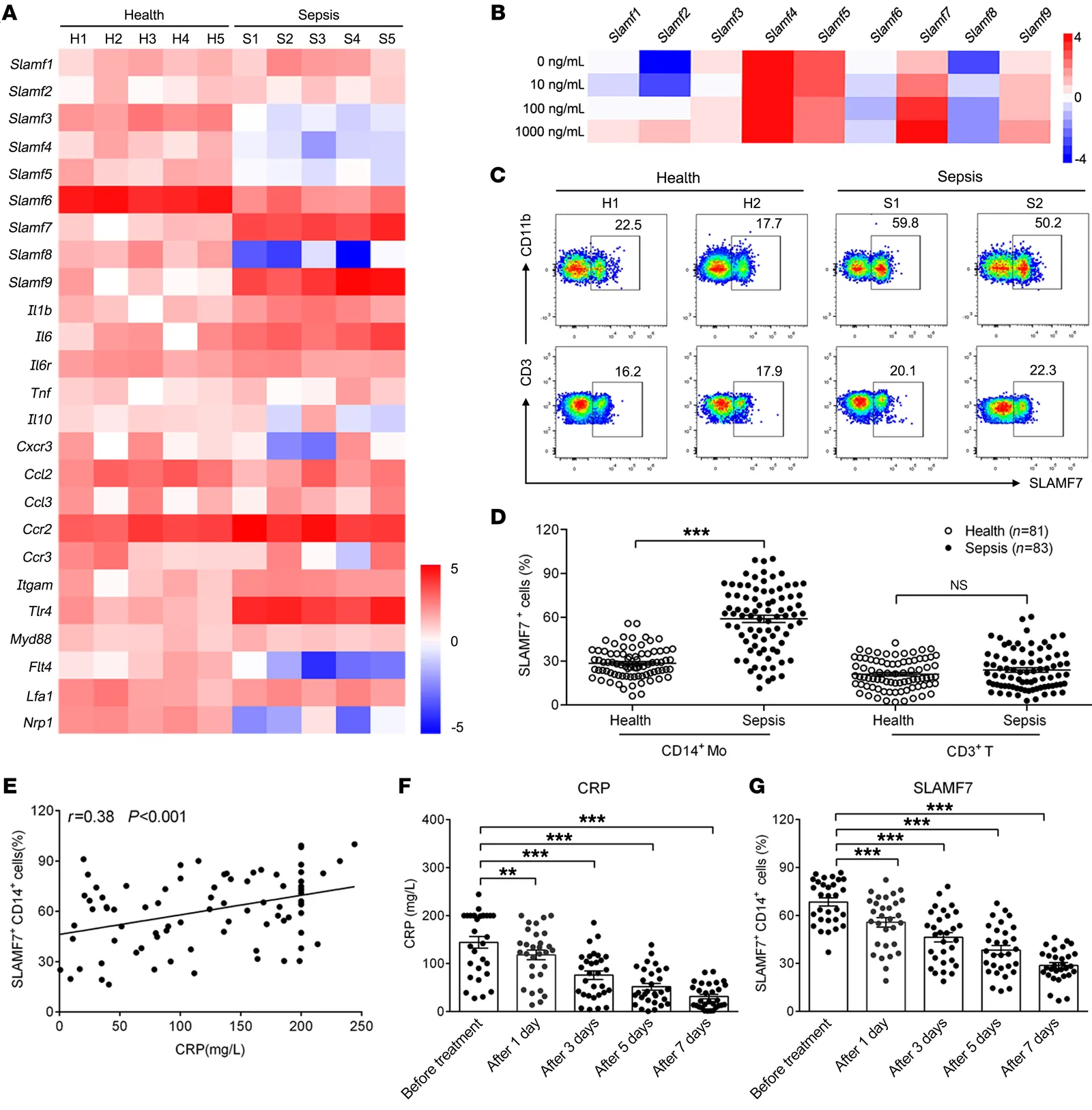
SLAMF7 expression is associated with sepsis. (**A**) Heatmap depicting mRNA expression levels of inflammation-related molecules in PBMCs from patients with sepsis (S1–S5) were determined by quantitative real-time PCR and compared with levels in healthy donors (H1–H5). The fold change for each gene was normalized to β-actin expression. (**B**) Expression of SLAMF members in human monocyte–derived macrophages after LPS stimulation for 12 hours at different doses. (**C** and **D**) Percentage of SLAMF7^+^ cells among CD11b^+^CD14^+^ or CD3^+^ cell subsets from human healthy donors (*n* = 81) and patients with sepsis (*n* = 83). Mo, monocytes. (**E**) Correlation between CRP and SLAMF7^+^ monocyte frequencies in patients with sepsis. (**F**) Protein levels of CRP in the serum of patients with sepsis were analyzed by ELISA before clinical treatment or 1, 3, 5, and 7 days after treatment. (**G**) The percentage of SLAMF7^+^ cells among CD14^+^ subsets was analyzed by flow cytometry before treatment and 1, 3, 5, and 7 days after treatment. Data represent the mean ± SEM from at least 3 independent experiments. **P < 0.01 and *** *P* < 0.001, by 2-tailed, unpaired Student’s *t* test (**D**), Spearman’s correlation (**E**), and 1-way ANOVA (**F** and **G**).

To evaluate the inflammatory mediators involved in TLR signaling during sepsis, we performed quantitative PCR to profile the gene expression of cytokines, chemokines, growth factors, and immune receptors in PBMCs from patients with sepsis (*n* = 5) and healthy individuals (*n* = 5). Consistent with previous studies (24), our results showed the induction of proinflammatory genes, including IL-6 (*Il6*) and IL-β (*Il1b*). In addition, multiple inflammation-related receptors, such as TLR4 and integrin subunit α M (*Itgam*, also called *Cd11b*) were induced in PBMCs from patients with sepsis. In the gene screen, we analyzed the expression of SLAMFs, which have not, to our knowledge, been studied in sepsis before. Interestingly, expression levels of *Slamf7* and *Slamf9* were substantially elevated, whereas *Slamf3*, *Slamf4*, *Slamf5*, *Slamf6*, and *Slamf8* expression levels were downregulated (Figure 1A). Since monocytes and macrophages are the major source of inflammatory cytokines involved in sepsis (25), we isolated human monocytes from healthy donors and stimulated the cells with different doses of endotoxin (LPS), followed by the detection of SLAMF expression. We found that the expression levels of *Slamf2*, *Slamf7*, *Slamf8*, and *Slamf9* were all increased, among which *Slamf7* expression was LPS dose dependent (Figure 1B).

Subsequently, we detected the expression of SLAMF7 protein in patients with sepsis by flow cytometry (Supplemental Figure 1; supplemental material available online with this article; https://doi.org/10.1172/JCI150224DS1). The results showed that the expression of SLAMF7 in CD11b^+^CD14^+^ monocytes, but not CD3^+^ T cells, was significantly increased in patients with sepsis (*n* = 83) (Supplemental Tables 1 and 2) compared with expression levels in healthy donors (*n* = 81) (Supplemental Table 1 and Figure 1, C and D). Then, we investigated whether SLAMF7 expression specificity correlated with age, sex, infection type, and disease complications. As a result, we found that SLAMF7 expression was not related to the above factors and did not differed among the groups (Supplemental Figure 2, A–D). Interestingly, data showed that the percentage of SLAMF7^+^ monocytes was strongly correlated with serum concentrations of C-reactive protein (CRP) (*r* = 0.38) (Figure 1E), which is a commonly used marker for inflammation and disease progress in sepsis (26, 27). More important, we observed positive correlations between the percentage of SLAMF7^+^ monocytes and the patients’ SOFA (*r* = 0.298) or SAPSII (*r* = 0.251) disease severity scores (Supplemental Figure 2, E and F). We thus evaluated the alternation of SLAMF7 expression in the progress of sepsis. We collected a series of blood samples from patients with sepsis on their ICU admission day (day 0; patients were diagnosed with sepsis and admitted to the ICU on the same day) and 1, 3, 5, and 7 days after treatment to detect the expression of SLAMF7. The results showed that the serum CRP concentration (Figure 1F) and the percentage of peripheral SLAMF7^+^CD14^+^ cells (Figure 1G) had both gradually decreased from the ICU admission day to day 7 after treatment. Overall, our results showed that SLAMF7 expression on monocytes was elevated in sepsis patients and was associated with sepsis progress, suggesting a possible connection between SLAMF7 and sepsis.

### SLAMF7 expression is induced by TLR/NF-κB signaling in monocytes and macrophages.

**Figure 2:**
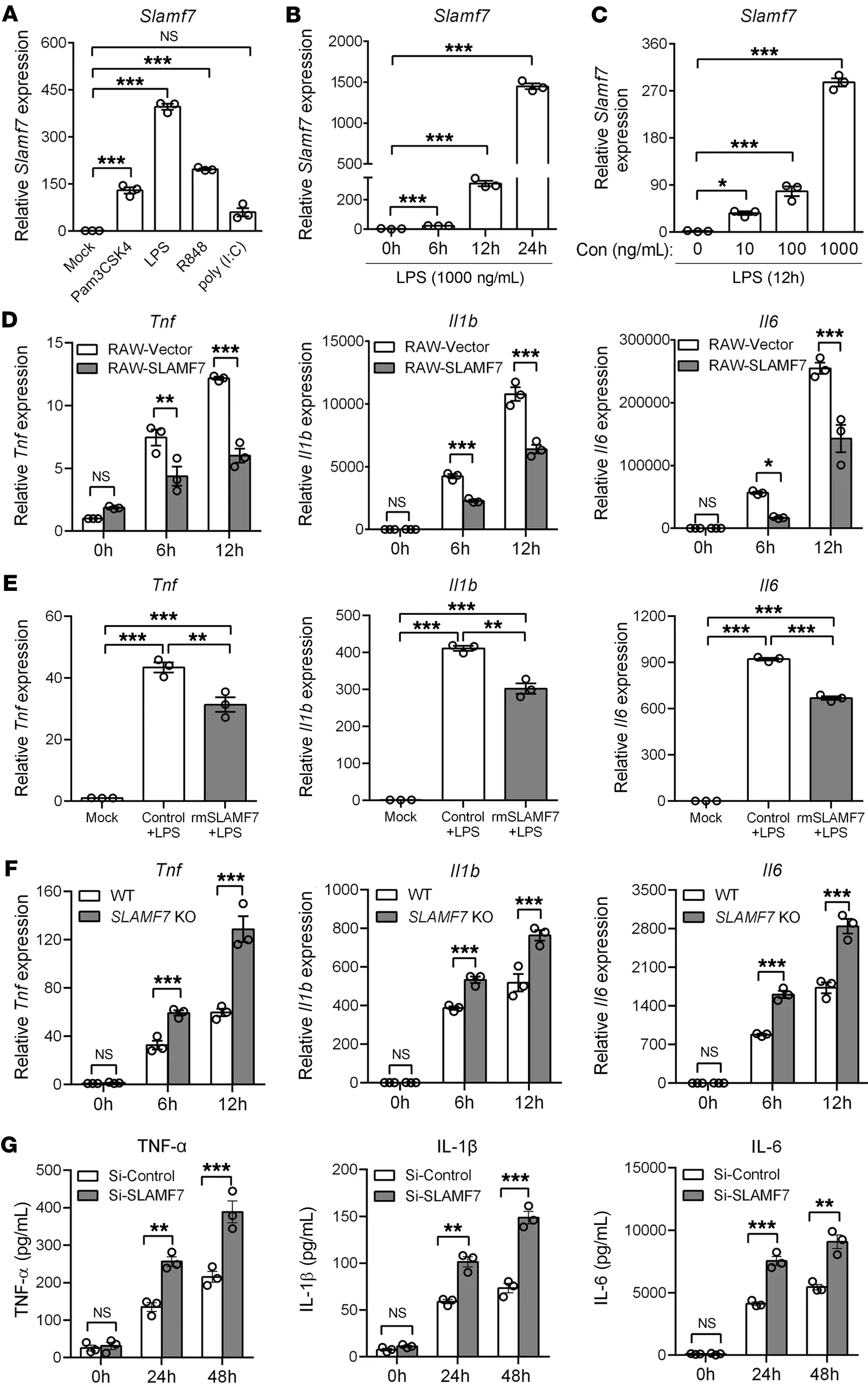
SLAMF7 negatively regulates the production of proinflammatory cytokines. (**A**) mRNA levels of *Slamf7* in RAW264.7 cells were examined by real-time PCR after stimulation with Pam3Csk4 (TLR1/-2 ligand), LPS (TLR4 ligand), R848 (TLR7/8 ligand), or poly (I:C) (TLR3 ligand) for 24 hours. (**B** and **C**) mRNA levels of *Slamf7* were examined in RAW264.7 cells after LPS treatment at the indicated time point (**B**) and concentration (Con) (**C**). (**D**) RAW264.7 cells stably expressing SLAMF7 (RAW-SLAMF7) and control RAW-vector cells were constructed. mRNA levels of *Tnf*, *Il1b*, and *Il6* in RAW-SLAMF7 cells versus RAW vector cells were analyzed 0, 6, and 12 hours after LPS stimulation. (**E**) Gene expression of *Tnf*, *Il1b*, and *Il6* in BMDMs before treatment with rmSLAMF7 protein (1 μg/mL) versus control (0.9% NaCl), 6 hours after LPS stimulation. (**F**) Gene expression of *Tnf*, *Il1b*, and *Il6* in WT and SLAMF7-KO BMDMs after LPS stimulation at 0, 6, and 12 hours. (**G**) Protein levels of TNF-α, IL-1β, and IL-6 in the supernatant of BMDMs transfected with SLAMF7 siRNA (Si-SLAMF7) or negative control siRNA (Si-Control), followed by LPS stimulation for 24 and 48 hours. Data represent the mean ± SEM from at least 3 independent experiments. **P < 0.01 and ***P < 0.001, by 2-tailed, unpaired Student’s *t* test (**D**, **F**, and **G**) and 1-way ANOVA (**A**–**C** and **E**).

Upon the recognition of ligands from pathogens, TLRs (except TLR3) initiate the MyD88-dependent pathway for NF-κB activation, which has been linked to the initiation and amplification of inflammation (28). We next examined whether TLR/MyD88/NF-κB signaling activation contributed to SLAMF7 expression. Using different TLR ligands to stimulate a RAW264.7 macrophage-like cell line, we observed that all of the TLR ligands except poly(I:C) induced *Slamf7* mRNA expression (Pam3Csk4 for TLR1/-2, LPS for TLR4, R848 for TLR7/-8, poly(I:C) for TLR3), among which LPS induced the maximal change in *Slamf7* expression compared with the other ligands (Figure 2A). Furthermore, we found that *Slamf7* mRNA expression was increased in mouse bone marrow–derived macrophages (BMDMs) and human CD14^+^monocytes after LPS stimulation (Supplemental Figure 3A). Data showed that LPS treatment (Figure 2, B and C) and *P*. *aeruginosa* (a common pathogenic bacterium in sepsis) infection (Supplemental Figure 3, B and C) induced the expression of *Slamf7* in both RAW264.7 cell lines in a time- and dose-dependent manner. Subsequently, we measured SLAMF7 expression in *TLR4*-KO BMDMs to explore whether its expression was TLR4 dependent. Unsurprisingly, we found that LPS did not increase *Slamf7* expression in *TLR4*-KO BMDMs (Supplemental Figure 3D). However, *Slamf7* expression was not entirely abolished in *TLR4*-KO BMDMs compared with expression in WT BMDMs upon infection with *P*. *aeruginosa* (Supplemental Figure 3E), which can also be recognized by TLR1/-2/-5/-6/-9 receptors in addition to TLR4 (29). Thus, we concluded that SLAMF7 expression on macrophages was induced by TLRs that included but were not limited to TLR4. As MyD88 is a known adaptor for the transmittion of downstream TLRs signals (30), we next investigated whether it regulated the expression of SLAMF7. After knockdown of MyD88 by siRNA, *Slamf7* expression was inhibited after LPS stimulation (Supplemental Figure 3, F and G). Meanwhile, sequence analysis with the JASPAR program predicted several potential NF-κB and activator protein 1–binding (AP-1–binding) sites in the promoter region of *Slamf7* (Supplemental Figure 3H), indicating the possible regulation of NF-κB and AP-1 during SLAMF7 transcription. As expected, the transcriptional level of *Slamf7* was almost diminished by the IκB kinase (IKK) pharmacological inhibitor BMS345541, followed by LPS stimulation. However, although MAPKs including ERK, JNK, and p38 lead to AP-1 activation (31), their inhibitors (SP600125 for JNK, SB203580 for p38, U0126 for ERK) unexpectedly had a limited effect on *Slamf7* transcript levels (Supplemental Figure 3I). Consistently, another chemical inhibitor, JSH-23, which blocks the translocation of the p65 subunit of NF-κB, also decreased *Slamf7* mRNA levels in both BMDMs and RAW264.7 cells (Supplemental Figure 3, J and K). These data suggested that TLR/MyD88/NF-κB signaling promoted SLAMF7 expression in monocytes and macrophages.

### SLAMF7 negatively regulates proinflammatory cytokine production in macrophages.

Deaths occurring in the early phase of sepsis are largely due to the cytokine storm and inflammation-induced multiorgan failure (1, 32–34). As the main mediator engaged in TLR-triggered innate inflammatory responses, macrophages participate in inflammation by releasing a large amount of proinflammatory cytokines (35). We then investigated the role of SLAMF7 in macrophage-mediated cytokine production. We constructed a RAW264.7 cell line steadily overexpressing SLAMF7 by lentivirus transfection (RAW-SLAMF7) (Supplemental Figure 4A). We observed reduced levels of proinflammatory cytokines (*Tnf*, *Il1b*, and *Il6*) in RAW-SLAMF7 cells versus RAW-vector cells (RAW264.7 cells with vector lentivirus transfection) induced by LPS (Figure 2D and Supplemental Figure 4B). To confirm these observations, BMDMs were treated with recombinant mouse SLAMF7 (rmSLAMF7) protein, which acts as a self-ligand to activate SLAMF7 signals by targeting the extracellular domain of SLAMF7 (36), followed by the detection of cytokine levels. The results showed that rmSLAMF7 significantly decreased the expression levels of cytokines (Figure 2E). Consistent with this, we observed excessive proinflammatory cytokines expression in *SLAMF7*-KO BMDMs compared with expression levels in WT BMDMs (Figure 2F). Moreover, we knocked down SLAMF7 in theRAW264.7 cell line by transfecting an siRNA targeting SLAMF7 (Supplemental Figure 4C), and proinflammatory cytokines were upregulated in SLAMF7 siRNA–knockdown RAW264.7 cells (Figure 2G). To broaden the regulatory range of SLAMF7 from TLR4-dependent effects to a TLR-triggered inflammatory response, WT and *TLR4*-KO BMDMs were stimulated with LPS (Supplemental Figure 5A) or infected with *P*. *aeruginosa* (Supplemental Figure 5B) in the presence of rmSLAMF7 protein and then tested the proinflammatory cytokines. Consistent with our previous findings, we observed that in WT BMDMs, both LPS and *P*. *aeruginosa* significantly induced the expression of *Tnf*, *Il1b*, and *Il6*, and activation of SLAMF7 with rmSLAMF7 largely decreased the expression of these 3 cytokines (Supplemental Figure 5, A and B). Meanwhile, as expected, TLR4 KO in BMDMs impaired the expression of inflammatory cytokines induced by LPS stimulation (Supplemental Figure 5A). However, *P*. *aeruginosa* infection could still upregulate the expression of inflammatory cytokines in *TLR4*-KO BMDMs, and rmSLAMF7 treatment reduced these cytokines levels in *TLR4*-KO BMDMs (Supplemental Figure 5B), which indicated that SLAMF7-regulated inflammatory responses partly dependent on TLR4 activation. Thus, we proposed that SLAMF7 regulated antiinflammatory immune responses in both a TLR4-dependent and -independent manner. Moreover, given that SLAMF7 is reported to promote the phagocytosis of tumor cells by macrophages (19), we also investigated whether SLAMF7 regulates bacterial phagocytosis by macrophages, which is a pivotal process for inflammation-mediated bacterial elimination. We performed the phagocytosis assay with *P*. *aeruginosa* and *E*. *coli*, which are2 of the most common pathogenic bacteria in sepsis (32). The results showed that SLAMF7 KO did not affect the phagocytosis of *P*. *aeruginosa* or *E*. *coli* by BMDMs (Supplemental Figure 6). Taken together, these data suggested that SLAMF7 exerted an inhibitory effect on TLR-triggered inflammatory responses in macrophages.

### SLAMF7 suppresses TLR4-triggered inflammatory signaling by activating the phosphorylation of SHIP1.

**Figure 3:**
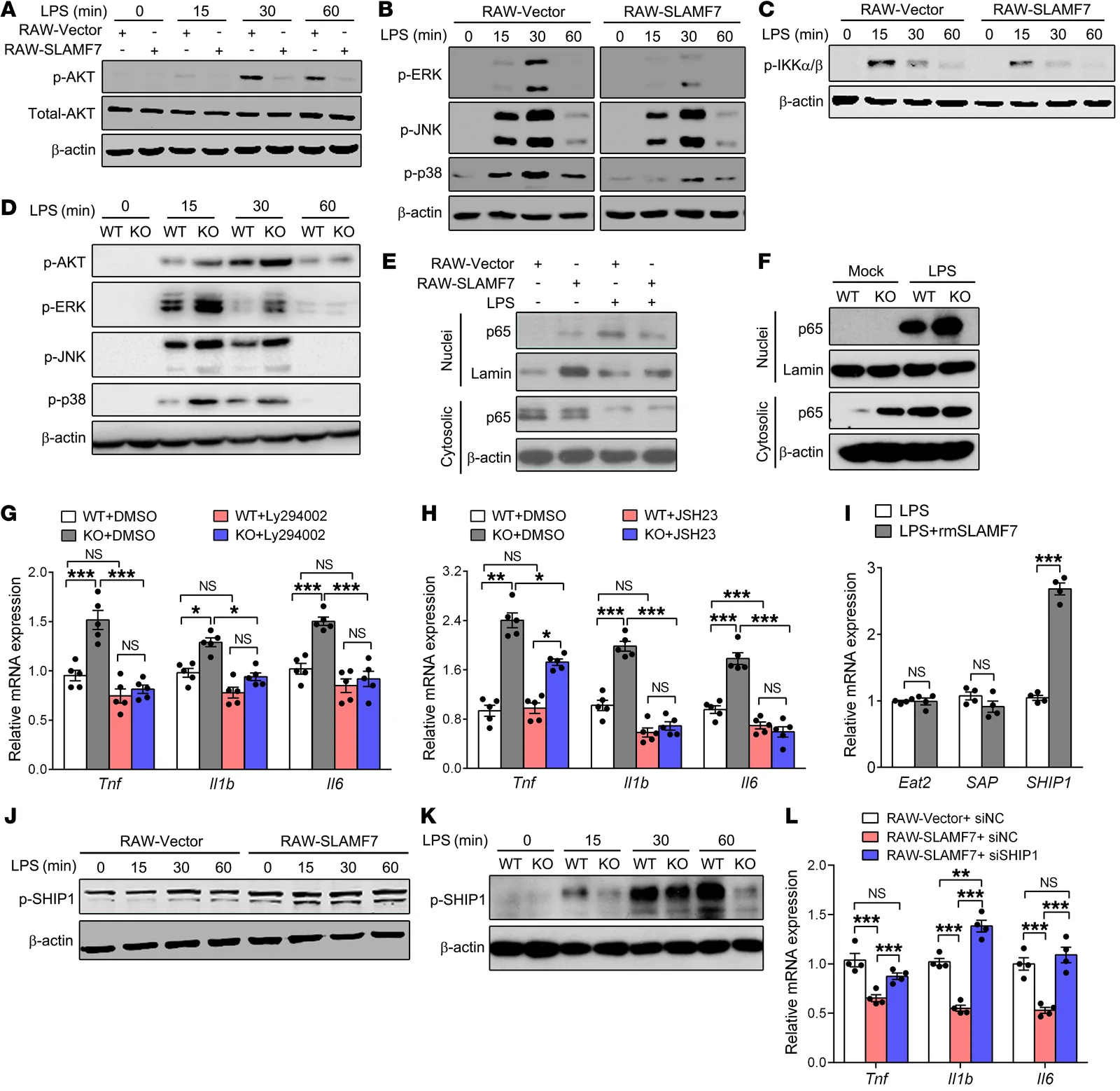
SLAMF7 attenuates MAPK/NF-κB signaling pathways by activating SHIP1. (**A**–**C**) Phosphorylation of AKT (**A**), MAPKs (ERK, JNK, and p38) (**B**), and IKKα/β (**C**) in RAW-SLAMF7 versus RAW-vector cells was examined by Western blotting after LPS stimulation at the indicated time points. (**D**) WT and SLAMF7-KO BMDMs were challenged with LPS for the indicated durations, followed by Western blotting to determine the phosphorylation of MAPKs and AKT. (**E** and **F**) Protein levels of the NF-κB p65 subunit in nuclei and the cytosolic fraction of LPS-treated RAW-vector versus RAW-SLAMF7 cells (**E**) and WT versus SLAMF7-KO BMDMs (**F**). (**G** and **H**) mRNA expression of *Tnf*, *Il1b*, and *Il6* in WT versus SLAMF7-KO BMDMs before treatment with an inhibitor targeting PI3K/Akt (Ly294002) (**G**) and NF-κB (JSH23) (**H**), followed by LPS stimulation for 6 hours. (**I**) mRNA expression of *Eat2*, *Sap*, and *Ship1* in BMDMs after stimulation with rmSLAMF7, followed by LPS stimulation. (**J** and **K**) Immunoblot (IB) analysis of p-SHIP1 in RAW-vector versus RAW-SLAMF7 cells (**J**) and WT versus SLAMF7-KO BMDMs (**K**) stimulated with LPS for 0, 15, 30, and 60 minutes. (**L**) mRNA expression of *Tnf*, *II1b*, and *Il6* in RAW264.7 cells after pretransfection with SHIP1 siRNA, followed by LPS stimulation for 6 hours. Data represent the mean ± SEM from at least 3 independent experiments. ns, not significant. *P < 0.05, **P < 0.01, and ***P < 0.001, by 2-tailed, unpaired Student’s *t* test (**I**) and 1-way ANOVA (**G**, **H**, and **L**).

As TLR4-triggered activation of PI3K/AKT, NF-κB, and MAPKs contributes to the production of inflammatory cytokines (35, 37), we next investigated the effect of SLAMF7 on these signaling pathways. We observed that overexpression of SLAMF7 reduced the phosphorylation of AKT (Figure 3A), ERK, p38 (Figure 3B), and IKKα/β (Figure 3C), but not JNK (Figure 3B). On the contrary, NF-κB (IKKα/β) and MAPK signaling (phosphorylated ERK [p-ERK], p-JNK, p-p38) were activated when SLAMF7 was knocked out in BMDMs (Figure 3D) or knocked down in RAW264.7 cells (Supplemental Figure 4A) after LPS stimulation. During the process of NF-κB activation, IKKα/β (IkB kinase) phosphorylates IkB and leads to its dissociation from NF-κB, thus allowing NF-κB to enter the nucleus and promote the expression of proinflammatory cytokine genes (38). We found that the amount of nuclear p65 translocated from the cytoplasm decreased in RAW-SLAMF7 cells versus RAW-vector cells (Figure 3E), but increased in *SLAMF7*-KO BMDMs versus WT BMDMs (Figure 3F). We therefore assessed the role of AKT and NF-κB signaling pathways in SLAMF7-regulated cytokine production by inhibiting AKT or NF-κB with Ly294002 (Figure 3G) or JSH23 (Figure 3H), respectively. We observed that treatment with either of these 2 inhibitors abrogated the upregulation of proinflammatory cytokines in *SLAMF7*-KO BMDMs (Figure 3, G and H). These results indicated that SLAMF7 inhibited inflammatory cytokine production by disturbing AKT and the NF-κB signaling pathway. To further elucidate how SLAMF7 affected signal transmission, we next investigated the adaptor for downstream signal transduction. It has been reported that SLAMFs transmit signals from the membrane into cytoplasm by utilizing SAPs, including EAT-2 and SAP (39). Nevertheless, a previous study reported that EAT-2 is barely expressed in primary human monocytes (16), suggesting the possibility that there are other adaptors for SLAMF7 in macrophages. Meanwhile, we noticed a potential signaling molecule that may connect SLAMF7 with downstream signals. A previous study reported the interaction of SLAMF7 with SHIP1 in EAT-2–deficient NK cells, which indicates the possibility of SHIP1 as an adaptor for SLAMF7 (18). We then used rmSLAMF7 to activate SLAMF7 signals in BMDMs and detected the expression of candidate adaptors for SLAMF7. Interestingly, the expression levels of *Eat-2* and *Sap* did not change after rmSLAMF7 stimulation, but *Ship1* mRNA expression displayed a 3-fold increase (Figure 3I). To confirm this observation, we measured the phosphorylation of SHIP1 (p-SHIP1), the active form of SHIP1, in RAW-SLAMF7 versus RAW-vector cells or in SLAMF7-KO versus WT BMDMs, followed by LPS stimulation for different durations. The data showed that the p-SHIP1 levels were increased in RAW-SLAMF7 versus RAW-vector cells (Figure 3J and Supplemental Figure 7B). In contrast, SLAMF7 KO attenuated p-SHIP1 levels in BMDMs (Figure 3K). Next, we explored the involvement of SHIP1 in SLAMF7-mediated signaling pathway transmission. We first transfected an siRNA targeting SHIP1 into RAW264.7 and confirmed the inhibitory effect of SHIP1 siRNA on the phosphorylation of SHIP1 (Supplemental Figure 7C). After the knockdown of SHIP1, we found that the inhibition of cytokines induced by SLAMF7 overexpression was reversed (Figure 3L). We thus demonstrated that SHIP1 knockdown disturbed SLAMF7 signal transduction. Studies suggest that SLAMF7 function is dependent on proto-oncogene tyrosine protein kinase Src and spleen tyrosine kinase (Syk) (15, 18). To identify the critical kinase downstream of SLAMF7 in macrophages, we analyzed the contribution of various signaling kinases to SLAMF7-mediated cytokine inhibition. The results showed that the pharmacological inhibitor BX795 targeting IKK-related kinase TANK-binding kinase 1 (TBK1) abrogated the inhibitory function of SLAMF7 in the inflammatory cytokine response in RAW264.7 cells, while the blockage of Src (PP2), Syk (R406), and Btk (ibrutinib) kinases had no effect (Supplemental Figure 7, D and E). Together, these data demonstrated that SHIP1 and NF-κB were involved in the SLAMF7-mediated suppression of TLR4-triggered inflammatory responses.

### SLAMF7 suppresses TRAF6 ubiquitination by interacting with SHIP1.

**Figure 4:**
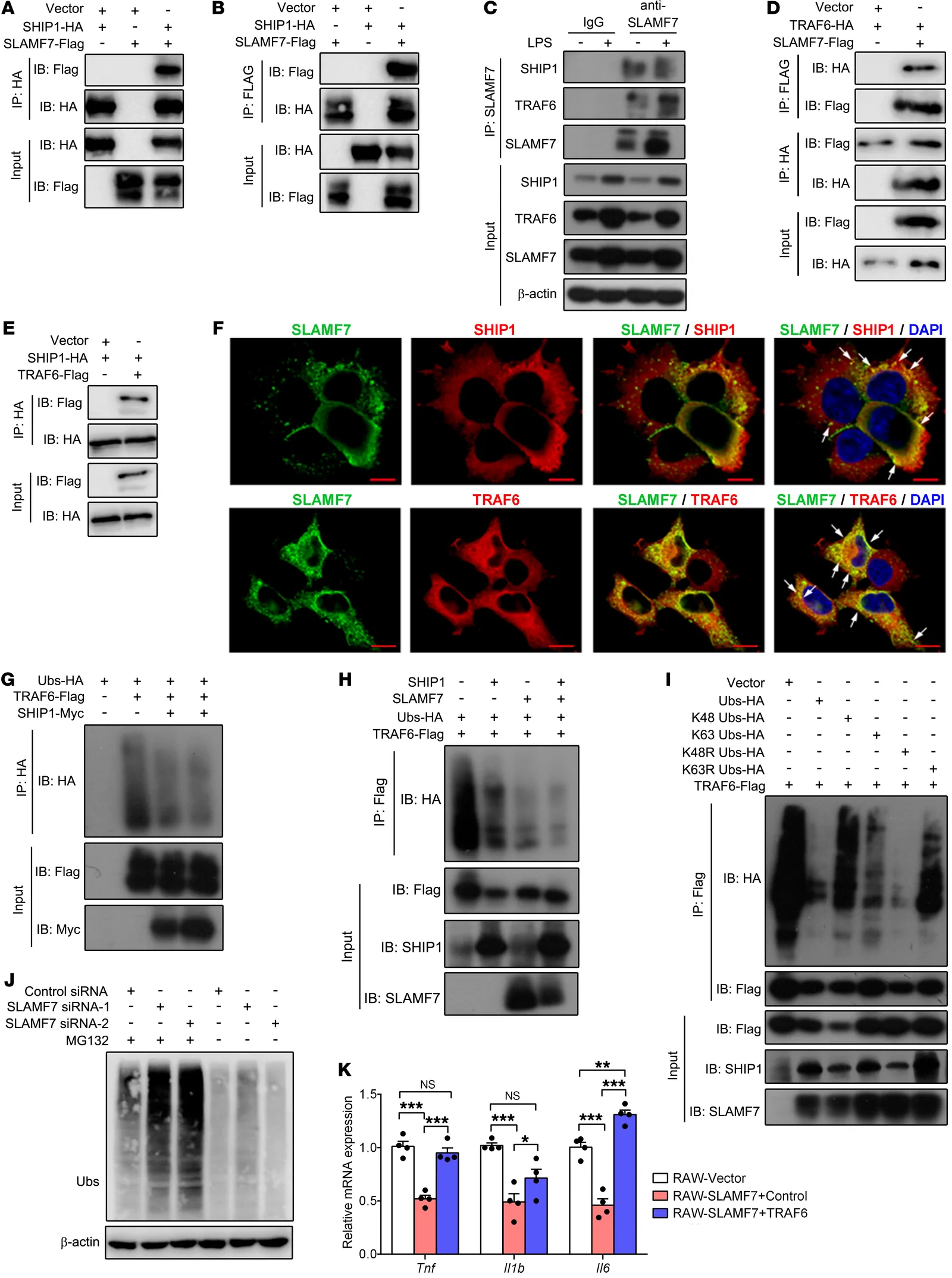
SLAMF7 cooperates with SHIP1 to inhibit TRAF6 K63 ubiquitination. (**A** and **B**) IP using anti-Flag or anti-HA antibodies from lysate of HEK 293T cells transfected with Flag-tagged SLAMF7 alone, or HA-tagged SHIP1. (**C**) Immunoassay of lysate of RAW264.7 cells stimulated with LPS, followed by IP with IgG or anti-SLAMF7 and IB analysis with anti-TRAF6 or anti-SHIP1. (**D** and **E**) IP using anti-Flag or anti-HA antibodies from lysate of HEK 293T cells transfected with HA-tagged TRAF6 and Flag-tagged SLAMF7 (**D**) or HA-tagged SHIP1 and Flag-tagged TRAF6 (**E**). (**F**) Confocal microscopy of HEK 293T cells cotransfected with Flag-tagged SLAMF7 and HA-tagged SHIP1 (top row) or HA-tagged TRAF6 (bottom row). White arrows indicate colocalization. Scale bars: 10 μm. (**G**) IB of TRAF6-Ubs precipitated with anti-HA antibodies from lysates of HEK 293T cells transfected with Flag-tagged TRAF6, HA-tagged Ubs, and Myc-tagged SHIP1. (**H**) IB analysis of TRAF6 ubiquitination from precipitation of lysates of 293T cells transfected with Flag-tagged TRAF6, HA-tagged ubiquitin with or without SLAMF7 and SHIP1. (**I**) IB of lysates of TRAF6 ubiquitination in HEK 293T cells transfected with HA-tagged ubiquitin, HA-tagged K48 ubiquitin, HA-tagged K63 ubiquitin, HA-tagged K48R ubiquitin, and HA-tagged K63 ubiquitin. (**J**) IB of TRAF6 ubiquitination of LPS-stimulated macrophages transfected with control siRNA or SLAMF7 siRNA, followed by LPS stimulation for 30 minutes, with or without MG132 treatment. (**K**) Relative mRNA expression of *Tnf*, *Il1b*, *Il6*, and *Il10* after transfection with a constructed TRAF6 plasmid or control plasmid. Data represent the mean ± SEM from at least 3 independent experiments. *P < 0.05, **P < 0.01, and ***P < 0.001, by 1-way ANOVA (**K**).

Previous studies reported that SLAMF7 can interact with SHIP1 to mediate inhibitory effects in the absence of the activated adaptor EAT-2 in NK cells (15). To investigate whether SLAMF7 can interact with SHIP1 directly, we transfected Flag-tagged SLAMF7 and HA-tagged SHIP1 into HEK 293T cells. Consistent with a previous study (18), IP analysis showed that SLAMF7 interacted with SHIP1 directly (Figure 4, A and B). TNF receptor–associated factors (TRAFs) play a central role in the regulation of inflammation through their activating role in TLR signaling pathways (40). Binding of LPS to TLR4 triggers the recruitment of MyD88 and IRAK1/-4, which then recruit TRAF6 to trigger downstream signaling (41). Here, we observed that activation of SLAMF7 significantly upregulated TRAF6 expression in LPS-stimulated BMDMs (Supplemental Figure 8). Endogenous IP analysis showed that SLAMF7, but not SHIP1, also interacted with TRAF6 (Figure 4C). Meanwhile, TRAF6 expression was upregulated by LPS stimulation, consistent with the expression of SHIP1 and SLAMF7 (Figure 4C). Exogenous IP showed the direct interaction of SLAMF7 with TRAF6 (Figure 4D), as well as the binding between SHIP1 and TRAF6 (Figure 4E). Furthermore, we confirmed that SLAMF7 colocalized with SHIP1 and TRAF6 by immunofluorescence (IF) assay (Figure 4F). These data suggested that SLAMF7 directly interacted with SHIP1 and TRAF6 to form a protein complex.

It has been demonstrated that SHIP1 reduces TRAF6 autoubiquitination and that autoubiquitination of TRAF6 is essential for subsequent NF-κB activation (42). We then explored whether SLAMF7 inhibited TRAF6 autoubiquitination via SHIP1. We transfected HA-tagged ubiquitination (Ubs-HA), Flag-tagged TRAF6, and Myc-tagged SHIP1 into HEK 293T cells to investigate the effect of SHIP1 on TRAF6 ubiquitination. The results showed that the ubiquitination of TRAF6 was reduced by SHIP1 (Figure 4G). Unexpectedly, we found that SLAMF7 also reduced TRAF6 ubiquitination, independently of SHIP1, but the inhibitory effect was enhanced by the cooperation with SHIP1 (Figure 4H). TRAF6 is a RING domain protein that catalyzes the synthesis of polyubiquitin chains linked through lysine 48 (K48) and lysine 63 (K63) of ubiquitin, and K63 is especially essential for the activation of the IKK and AKT signaling pathways (43, 44). We then sought to investigate the type of TRAF6 ubiquitin regulated by SLAMF7 and SHIP1. Through the lysine mutation of ubiquitin, we observed that SLAMF7 cooperated with SHIP1 to attenuate K63-linked, but not K48-linked, TRAF6 autoubiquitin (Figure 4I). Moreover, we found that total ubiquitination levels were increased after knockdown of SLAMF7 and that these levels increased further after treatment with a proteasome inhibitor (MG132), indicating that SLAMF7-induced ubiquitination inhibition was mediated by a proteasome degradation mechanism (Figure 4J). Finally, we found that SLAMF7-inhibited expression of *Tnf*, *Il1b*, and *Il6* was reversed by transfection with a TRAF6 plasmid, suggesting that TRAF6 was involved in the inhibition of cytokines by SLAMF7 (Figure 4K). Collectively, these results revealed that SLAMF7 interacted with TRAF6 and restricted its K63 autoubiquitination by cooperation with SHIP1 to inhibit inflammatory cytokine production.

### The interaction of SLAMF7 with TRAF6 and SHIP1 is dependent on the phosphatase domain of SHIP1.

**Figure 5:**
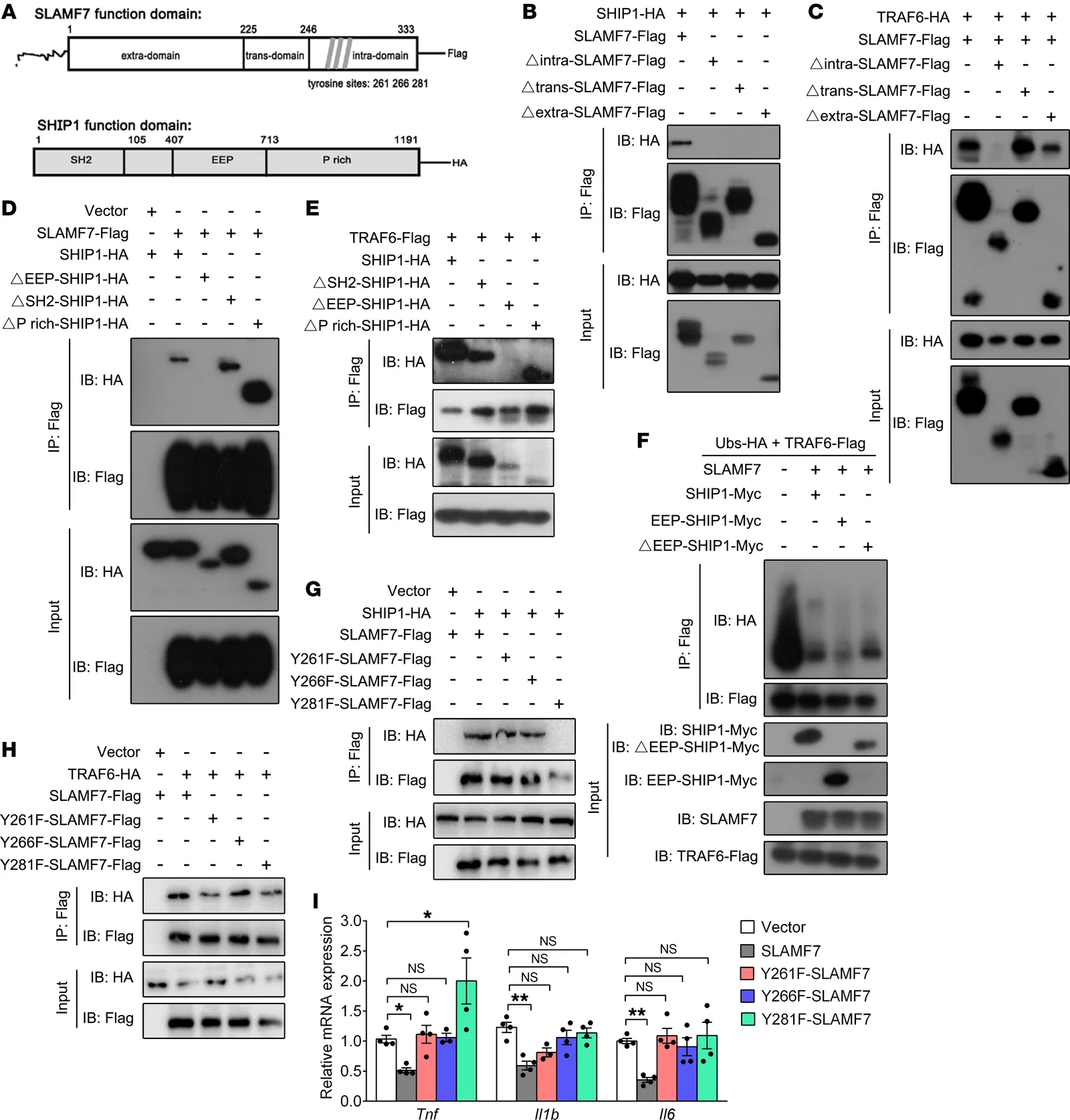
Binding of SLAMF7 to SHIP1 by tyrosine and the EEP domain is required for negative regulation of TLR responses. (**A**) Functional domains of SLAMF and SHIP1. (**B** and **C**) SLAMF7 plasmids lacking extracellular (Δextra), transmembrane (Δtrans), or intracellular (Δintra) domains were transfected into HEK 293T cells with SHIP1 (**B**) or TRAF6 (**C**) plasmid to determine the interactions. (**D** and **E**) IP was performed in HEK 293T cells cotransfected with SLAMF7 (**D**) or TRAF6 (**E**) plasmids as well as with full-length SHIP1 or SHIP1 plasmids lacking the SH2 domain (ΔSH2), the EEP domain (ΔEEP), and the P-rich domain (ΔP rich). (**F**) IB assay of endogenous ubiquitination of TRAF6 using anti-HA from lysate immunoprecipitated with anti-Flag from HEK 293T cells transfected with HA-tagged ubiquitin (Ubs-HA) or Flag-tagged TRAF6 (TRAF6-Flag), plus SLAMF7 alone or SLAMF7 plus EEP-HA or SLAMF7 plus ΔEEP-HA. (**G** and **H**) Tyrosine sites at amino acids 261, 266, and 281 of the SLAMF7 amino acid sequence, ignoring signal peptide were manually mutated to phenylalanine (Y→F), respectively. IP was performed in HEK 293T cells after the transfection of SHIP1 (**G**) or TRAF6 (**H**) plasmid with full-length SLAMF7 or SLAMF7 tyrosine mutations. (**I**) *Tnf*, *Il1b*, and *Il6* mRNA levels in RAW264.7 cells transfected with Y261F-, Y266F-, or Y281F-SLAMF7 plasmids, followed by LPS stimulation for 6 hours. Data represent the mean ± SEM from at least 3 independent experiments. *P < 0.05 and **P < 0.01, by 1-way ANOVA (**I**).

Next, we sought to determine the binding domains that are required for the interaction of SLAMF7 with SHIP1 and the suppression of TLR4-triggered inflammatory responses. SLAM family molecules contain an N-terminal extracellular domain, a single transmembrane domain, and a cytoplasmic tail (45). Unlike the other members, SLAMF7 has a relatively long cytoplasmic tail that contains 3 tyrosine phosphorylation sites (Figure 5A) (46). SHIP1 contains an amino-terminal Src homology 2 domain (SH2 domain) that binds preferentially to the sequence pY(Y/S/T) L(M/L), a centrally located phosphoinositol phosphatase domain (EEP domain) that selectively hydrolyzes the 5′-phosphate and a critical proline-rich C-terminus (P-rich domain) that binds a subset of SH3-containing proteins (Figure 5A) (47, 48). First, we generated a panel of domain deletion constructs of SLAMF7 (respectively deleting the intracellular, transmembrane, and extracellular domains) and performed a co-IP assay. The results showed that if SLAMF7 lacked any one of the domains, it failed to bind to SHIP1, which indicated that the interaction was dependent on the full-length SLAMF7 (Figure 5B). However, deletion of the intracellular domain of SLAMF7 rendered it unable to bind with TRAF6 compared with the other 2 truncated fragments and full-length SLAMF7, demonstrating that the interaction between SLAMF7 and TRAF6 was dependent on the intracellular domain of SLAMF7 (Figure 5C). In addition, we showed that the EEP domain of SHIP1 was responsible for the interaction with SLAMF7 (Figure 5D) or TRAF6 (Figure 5E). Moreover, we found that the EEP domain of SHIP1 substantially assisted SLAMF7 in inhibiting the autoubiquitination of TRAF6 (Figure 5F). However, SHIP1 without an EEP domain only partially reversed the inhibition, which suggested that SLAMF7 also inhibited TRAF6 autoubiquitination in an EEP domain–independent manner (Figure 5F).

As mentioned above, SLAMF7 has 3 tyrosine phosphorylation sites (Y261, Y266, and Y281) in the cytoplasmic domain (49, 50). To elucidate whether these 3 tyrosines mediate SLAMF7 signal transduction, we constructed SLAMF7 mutants with cytoplasmic tyrosines (Y) mutated to phenylalanines (F) (Y-to-F mutations) (Figure 5A). Through the IP assay, we found that SLAMF7 with Y281 mutation failed to bind to SHIP1, indicating that Y281 of SLAMF7 played a decisive role in SLAMF and SHIP1 interaction (Figure 5G). Furthermore, we showed that the interaction between SLAMF7 and TRAF6 was independent of Y261, Y266, and Y281 tyrosines (Figure 5H). Last, we investigated whether the tyrosine mutations in SLAMF7 resulted in impaired cytokine inhibition. RAW264.7 cells were respectively transfected with three SLAMF7 mutants to detect cytokine production. Interestingly, we found that mutated SLAMF7 failed to downregulate the expression of cytokines including *Tnf*, *Il1b*, and *Il6* (Figure 5I). Meanwhile, among 3 tyrosine mutations, Y281 mutation most significantly reversed the cytokine production (Figure 5I). On the basis of the above observations, we demonstrated that the tyrosines within the cytoplasmic domain of SLAMF7 were crucial for the inhibitory role of SLAMF7 in cytokine production.

### Activation of SLAMF7 rescues septic mice by inhibiting the inflammatory response.

**Figure 6:**
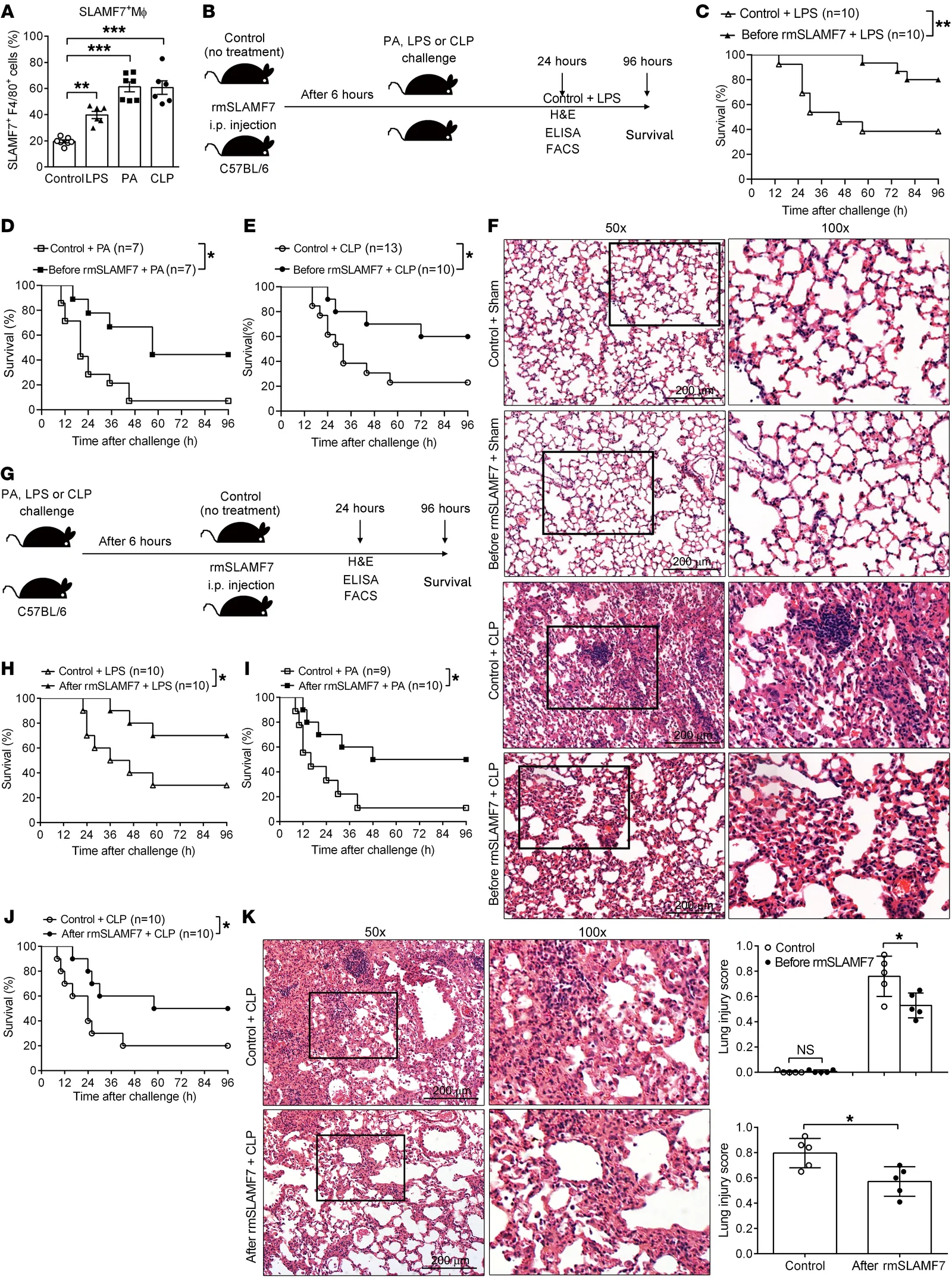
SLAMF7 protects against sepsis by inhibiting inflammation and lung injury. A sepsis model was established in C57BL/6 (B6) mice by i.p. injection with LPS (25 mg/kg) or *P*. *aeruginosa* (PA) (2 × 10^7^ CFU/kg) or by CLP surgery. (**A**) Percentage of SLAMF7^+^ macrophages in the PL was analyzed by flow cytometry. Mɸ, macrophages. (**B**–**F**) Mice were i.p. injected with rmSLAMF7 or vehicle control (0.9% NaCl), followed by the establishment of LPS, *P*. *aeruginosa*, or CLP sepsis 6 hours later. (**B**) Schematic of rmSLAMF7 administration prior to sepsis. (**C**–**E**) Survival rates of mice challenged with LPS (**C**), *P*. *aeruginosa* (**D**), or CLP (**E**). (**F**) H&E staining of lung sections was examined in rmSLAMF7 and vehicle control–treated mice 24 hours after CLP. Scale bars: 200 μm. (**G**–**K**) Mice were i.p. injected with LPS, *P*. *aeruginosa*, or CLP to establish sepsis models. Six hours after injection, rmSLAMF7 or vehicle control was i.p. administrated. (**G**) Schematic of rmSLAMF7 administration after sepsis induction. (**H**–**J**) Survival rates of mice after LPS (**H**), *P*. *aeruginosa* (**I**), or CLP (**J**) challenge. (**K**) H&E staining was performed 24 hours later to evaluate lung injury. Scale bars: 200 μm. Data represent the mean ± SEM from at least 3 independent experiments. *P < 0.05, **P < 0.01, and ***P < 0.001, by 1-way ANOVA (**A**), log-rank test (**C**–**E** and **H**–**J**), and 2-tailed, unpaired Student’s *t* test (**F** and **K**).

To investigate the role of SLAMF7 in sepsis in vivo, we established sepsis mouse models in C57BL/6 mice by LPS injection (endotoxemia model), *P*. *aeruginosa* infection (bacterial sepsis model), or cecal ligation and puncture (CLP) (polymicrobial sepsis model) (51). Consistent with the data from the patients with sepsis, the percentage of SLAMF7^+^F4/80^+^ macrophages in the peritoneal lavage (PL) was significantly elevated in LPS-, *P*. *aeruginosa*–, or CLP-induced septic mice (Figure 6A). To further confirm the involvement of SLAMF7 in sepsis progress, we activated SLAMF7 in vivo with rmSLAMF7 protein and observed the survival rates and lung histopathology of mice after sepsis induction (Figure 6B). The results showed that rmSLAMF7 pretreatment largely improved the survival of LPS- (Figure 6C), *P*. *aeruginosa*– (Figure 6D), or CLP-induced (Figure 6E) septic mice. Thus, SLAMF7 appeared to play an important role in reducing the mortality rate of septic mice. Moreover, mice pretreated with rmSLAMF7 protein displayed less lung injury and inflammatory cell infiltration in CLP- (Figure 6F), LPS- (Supplemental Figure 9A), or *P*. *aeruginosa*–induced (Supplemental Figure 9B) septic mice. In addition, rmSLAMF7 reduced the number of TUNEL^+^ apoptosis cells in lung tissues from septic mice (Supplemental Figure 9C). Next, to explore the therapeutic effect of rmSLAMF7 protein, we generated a sepsis model and subsequently treated mice with rmSLAMF7 protein to observe survival rates and lung histopathology (Figure 6G). Similarly, rmSLAMF7 treatment after sepsis significantly increased the survival of LPS- (Figure 6H), *P*. *aeruginosa*– (Figure 6I), or CLP-induced (Figure 6J) septic mice. Moreover, rmSLAMF7 protein alleviated lung injury and inflammatory cell infiltration in CLP septic mice (Figure 6K). To explore whether SLAMF7 regulate the production of proinflammatory cytokines induced by sepsis in vivo, we determined the concentrations of TNF-α, IL-1β, and IL-6 in serum and supernatants of liver, lung, and PL. As expected, we observed significant reductions in the levels of TNF-α, IL-1β, and IL-6 in rmSLAMF7-treated CLP- (Supplemental Figure 10A) and LPS-induced septic mice (Supplemental Figure 10B). These results suggested that SLAMF7 alleviated systemic inflammation and protected mice against sepsis. Moreover, we found that the activation of SLAMF7 decreased the percentages of TNF-, IL-1β–, or IL-6–producing macrophages (Supplemental Figure 10C), and there was no difference in the percentage of F4/80^+^ macrophages from PL in rmSLAMF7-treated mice compared with control mice (Supplemental Figure 10D), indicating that SLAMF7 downregulated cytokine production of macrophages instead of reducing macrophage numbers. Thus, we demonstrated that SLAMF7 attenuated the in vivo inflammatory response in sepsis.

### KO of SLAMF7 contributes to the exacerbated inflammatory response in sepsis.

**Figure 7:**
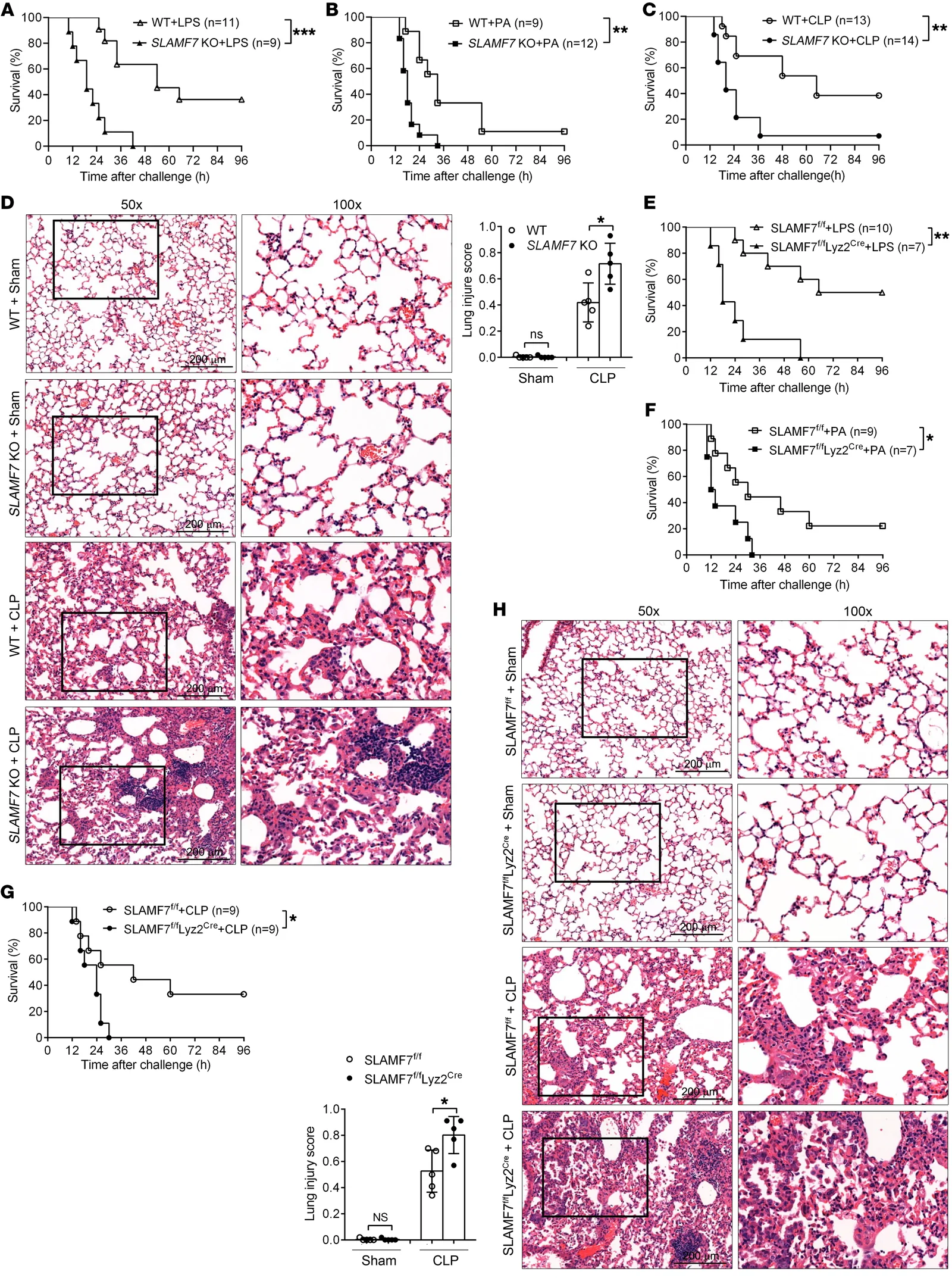
SLAMF7 deficiency exacerbates sepsis by aggravating inflammation and lung damage. WT mice and SLAMF7-KO mice were subjected to LPS- (25 mg/kg), *P*. *aeruginosa*– (2 × 10^7^ CFU/kg), or CLP-induced sepsis. (**A**–**C**) Survival curves were calculated after LPS (**A**), *P*. *aeruginosa* (**B**), or CLP (**C**) challenge. (**D**) H&E staining of lung sections was examined 24 hours after CLP. Scale bars: 200 μm. (**E**–**H**) LPS- (25 mg/kg), *P*. *aeruginosa*– (2 × 10^7^ CFU/kg), or CLP-induced sepsis models were established in control (SLAMF7^fl/fl^) and SLAMF7 conditional-KO mice (SLAMF7^fl/fl^ Lyz2^Cre^). (**E**–**G**) Survival rates of mice after LPS (**E**) or *P*. *aeruginosa* (**F**) injection or CLP surgery (**G**). (**H**) Twenty-four hours after CLP surgery, H&E staining was performed to assess injury and inflammatory infiltration into lung tissues. Scale bars: 200 μm. Data represent the mean ± SEM and represent 3 individual experiments *P < 0.05, **P < 0.01, and ***P < 0.001, by log-rank test (**A**–**C** and **E**–**G**) and 2-tailed, unpaired Student’s *t* test (**D** and **H**).

To fully reveal the in vivo function of SLAMF7 in the development of sepsis, we further established LPS, *P*. *aeruginosa*, and CLP sepsis mouse models with WT and *SLAMF7*-KO mice. We found that the mortality of *SLAMF7*-KO mice was higher than that of WT mice (Figure 7, A–C). Furthermore, we observed greater infiltration of inflammatory cells and lung structural damage in *SLAMF7*-KO mice compared with WT mice after CLP (Figure 7D). These data suggested that SLAMF7 played a protective role in septic mice by improving survival and reducing pathological damage. In addition, we demonstrated that *SLAMF7*-KO mice displayed a remarkably elevated level of inflammatory cytokines (TNF-α, IL-1β, and IL-6) compared with WT mice after CLP (Supplemental Figure 11A) or LPS treatment (Supplemental Figure 11B). We also observed that SLAMF7 KO promoted the secretion of TNF, IL-1β, or IL-6 by macrophages (Supplemental Figure 11C) without affecting the percentage of macrophages present after CLP (Supplemental Figure 11D), which is consistent with our observations from the SLAMF7 activation experiments. These results indicated that SLAMF7 was required for the inhibition of excessive inflammation in sepsis.

To demonstrate that SLAMF7 exerts its function in sepsis through macrophages, we depleted macrophages in WT and *SLAMF7*-KO septic mice through clodronate liposomes (Supplemental Figure 12A) and then established a CLP model (52). Consistent with previous studies (53, 54), macrophage depletion reduced survival rates (Supplemental Figure 12B) and increased the bacterial load in the PL and blood of septic mice (Supplemental Figure 12C). However, there were no differences in survival rates or bacterial load between WT and *SLAMF7*-KO septic mice after macrophage deletion (Supplemental Figure 12, B and C). Furthermore, we detected no differences in IL-1β, IL-6, or TNF-α levels between WT and *SLAMF7*-KO mice after macrophage deletion (Supplemental Figure 12D), indicating the crucial role of macrophages in SLAMF7-mediated inflammation inhibition during sepsis.

To directly determine the role of macrophage SLAMF7 in sepsis, we generated a conditional SLAMF7^fl/fl^ Lyz2^Cre^ mouse by crossing mice that had loxP-flanked alleles of the SLAMF7 exon (SLAMF7^fl/fl^) with mice that had transgenic expression of Cre recombinase driven by the gene encoding Lyz2 (Lyz2^Cre^), in which SLAMF7 is specifically deleted in macrophages. We then established LPS-, *P*. *aeruginosa*–, and CLP-induced sepsis models using SLAMF7^fl/fl^ and SLAMF7^fl/fl^ Lyz2^Cre^ mice. The results consistently showed that SLAMF7^fl/fl^ Lyz2^Cre^ mice had higher mortality rates than did SLAMF7^fl/fl^ mice after LPS (Figure 7E), *P*. *aeruginosa* (Figure 7F), or CLP (Figure 7G) challenge. Meanwhile, we observed a greater amount of infiltration of inflammatory cells and lung structural damage in SLAMF7^fl/fl^ Lyz2^Cre^ mice than in SLAMF7^fl/fl^ mice after CLP (Figure 7H). These data suggested that the SLAMF7-conferred protection against sepsis was dependent on macrophages and that macrophages were a key immune cell subset in SLAMF7-mediated inflammation inhibition during sepsis.

### SLAMF7 does not affect sepsis-induced immunosuppression.

In the late stage of sepsis, patients usually undergo an immunosuppression stage, in which immune defense activities and proinflammatory responses are inhibited, while antiinflammatory reactions are active (32, 55). Patients with this immunosuppression status are more susceptible to pathogens and usually die of secondary infections (56, 57). To investigate whether SLAMF7 is involved in the regulation stage of immunosuppression, we generated mouse models of sepsis immunosuppression according to previous studies (58–60). Lethal and moderate CLP models were respectively established with or without treatment with the antibiotic ertapenem. After 15 days (~30%–70% of the mice among the groups had survived) (Supplemental Figure 13A) (60), mice that survived sepsis were challenged with *P*. *aeruginosa* to mimic a secondary infection. The results showed that the mice that survived CLP were in the immunosuppression state, as reflected by increased bacterial counts compared with sham-treated mice after secondary *P*. *aeruginosa* infection (Supplemental Figure 13B). We first determined the expression of SLAMF7 at the different time points after CLP. After antibiotic treatment, SLAMF7 expression in macrophages was decreased 10 days after CLP (Supplemental Figure 13C), consistent with downregulated expression of SLAMF7 in patients with sepsis after treatment (Figure 1). However, the secondary infection with *P*. *aeruginosa* did not upregulate SLAMF7 expression (Supplemental Figure 13C), which was different from what we observed upon the primary infection with *P*. *aeruginosa* (Figure 6A). To explored the role of SLAMF7 in the mouse model of sepsis-induced immunosuppression, we established a lethal CLP model with antibiotics in WT and *SLAMF7*-KO mice, followed by infection with *P*. *aeruginosa* 15 days later (Supplemental Figure 13D). We found that KO of SLAMF7 did not affect the survival rate of the mice with sepsis-induced immunosuppression (Supplemental Figure 13E). Meanwhile, we observed no difference in *P*. *aeruginosa* bacterial burden in the lungs of WT and *SLAMF7*-KO mice after secondary *P*. *aeruginosa* infection (Supplemental Figure 13F), indicating the dispensable role of SLAMF7 in the immunosuppression stage of sepsis. To further verify these observations, WT and *SLAMF7*-KO mice were subjected to another immunosuppression model, in which moderate CLP was performed without the use of antibiotics, followed by the rechallenge of *P*. *aeruginosa* infection (Supplemental Figure 13G). Consistently, we observed no differences in survival rates or bacterial burden between WT and *SLAMF7*-KO mice after *P*. *aeruginosa* challenge (Supplemental Figure 13, H and I). Therefore, we concluded that SLAMF7 primarily exerted an antiinflammatory role in the early acute phase of sepsis, but had a limited effect on sepsis-induced immunosuppression, which merits further investigation.

## Discussion

SLAMF7 is an immunoglobulin superfamily receptor that plays a critical role in NK cell cytolytic activity in tumors (61). In this study, we demonstrated that SLAMF7 was inducibly expressed on macrophages in response to TLR ligands and bacterial infection and that it interacted with SHIP1 and TRAF6 to attenuate MAPK and NF-κB signaling–mediated proinflammatory cytokine production (Supplemental Figure 14). Finally, a rmSLAMF7 peptide agonist or genetic KO of SLAMF7 in mice demonstrated that SLAMF7 protected against lethal sepsis and endotoxemia by suppressing inflammatory responses. Overall, this study demonstrated a negative regulatory role of SLAMF7 in sepsis-induced inflammation and revealed the signal transduction mechanism involved in this process, which may provide new sights into the therapeutic targets for sepsis.

As a surface receptor with broad expression on immune cells and MM tumor cells (62), SLAMF7 is gradually becoming a promising checkpoint in tumor immunotherapy. In several clinical trials, the SLAMF7 monoclonal antibody elotuzumab has shown a great advantage in combination with other therapies for the treatment of relapsed or refractory multiple myeloma (RRMM) (63-65). Meanwhile, SLAMF7-directed chimeric antigen receptor–modified (CAR-modified) T cells have been used to treat tumors (66). SLAMF7 is also critical for phagocytosis of hematopoietic tumor cells via Mac-1 integrin (19). In 2013, PDL241, a novel humanized monoclonal antibody against SLAMF7 that inhibits IgM production from plasmablasts and plasma cells, showed beneficial effects on joint-related parameters (67). Moreover, recent studies also reported the role of SLAMF7 in HIV infection (22) and poly I:C/d-galactosamine–induced hepatitis (68). However, the regulatory function of SLAMF7 during infectious disease has not been clarified.

Here, we provide evidence that SLAMF7 was engaged in the progress of sepsis. SLAMF7 expression was significantly elevated on peripheral monocytes of patients with sepsis and varied with the disease severity. Furthermore, we found a positive correlation between SLAMF7 expression levels and disease severity indicators in patients. Importantly, we identified that SLAMF7 could reduce inflammatory cytokine (TNF-α, IL-1β, IL-6) secretion in vivo and in vitro by inhibiting the AKT, NF-κB, and MAPK signaling pathways. Furthermore, activation of SLAMF7 signal by recombined protein rescued mice from lethal sepsis, contributing to a better overall survival, while SLAMF7 KO accelerated sepsis by amplifying inflammatory responses. Besides, previous studies indicated that SLAMF7 acts as a phagocytic receptor to promote macrophage phagocytosis of hematopoietic tumor cells (19). However in the present study, we found that SLAMF7 had no effect on the phagocytosis of *P*. *aeruginosa* or *E*. *coli* by macrophages. Of note, although SLAMF7 regulated inflammation and organ damage in the acute phase of sepsis, we found that SLAMF7 had no effect on immunosuppression-caused deaths resulting from secondary *P*. *aeruginosa* infection. Moreover, SLAMF7 did not affect the bacterial burden in vivo, which mostly contributed to mortality of the mice with sepsis-induced immunosuppression (56, 57). Thus, we revealed the protective role of SLAMF7 in sepsis through its inhibition of the inflammatory response.

Previous studies have documented that several SLAMF members participate in infectious diseases (69, 70). Evidence for direct interaction of SLAMF1 with *E*. *coli* outer membrane porins C (OmpC) and OmpF has been shown in a cell-based luciferase reporter assay (71), while SLAMF2 is implicated in the recognition of nonopsonized *E*. *coli* via caveolae, resulting in phagocytosis by mast cells (72). Moreover, SLAMF1 also engages with measles virus hemagglutinin (MV-H), which provides the templates for antiviral drug design (73). Unlike some SLAMFs, SLAMF7 is a self-ligand receptor that recognizes itself to regulate NK cell cytolytic activity in tumors (36). To date, the definite function and mechanisms of SLAMF7 in infectious disease are still unclear. It has previously been reported that increased SLAMF7 expression in macrophages alleviates corneal inflammation caused by *P*. *aeruginosa* (23). In addition, chronic HIV-1 infection increases SLAMF7 expression, whereas SLAMF7 negatively regulates IFN-α–mediated CXCL10 production to inhibit the induction of inflammatory monocyte subsets (22). In this study, we demonstrated the negative modulatory role of SLAMF7 in the inflammatory response during sepsis. A previous report revealed that SLAMF7 expression is induced on LPS-activated monocytes via the NF-κB/PI3K pathway and that cross-linking of SLAMF7 decreases the secretion of TNF-α and IL-12p70 by LPS-activated monocytes (16). In our study, we demonstrated that NF-κB, but not AP-1, promoted the expression of SLAMF7 transcripts in murine macrophages. Moreover, we demonstrated in this study that SLAMF7 negatively regulated cytokine production, which was dependent on the activation of AKT and NF-κB signaling pathways. Extensive research on the signal transduction pathways has uncovered a connection between the PI3K/AKT and NF-κB pathways. AKT has been reported to contribute to the direct phosphorylation of the amino acid residue T23 on IKK, thereby leading to activation of NF-κB (74). In our results, knockdown of SLAMF7 promoted the activation of the AKT and NF-κB pathways and increased the phosphorylation of ERK, JNK, and p38. Blockade of the PI3K/AKT and NF-κB pathways by an inhibitor abolished the inhibition of cytokine production by SLAMF7, which indicated that SLAMF7 exerted antiinflammatory functions via these 2 signaling pathways.

SLAMF7 is reported to positively regulate NK cell function through the adaptor EAT-2, but not SAP. However, in the absence of EAT-2, SLAMF7 potently inhibits NK cell function by recruiting the inhibitory effectors SHP-1, SHP-2, SHIP1, Csk, and Fyn (18). Thus, SLAMF7 exerts activating or inhibitory effects on immune cells depending on the cellular context and the availability of effector proteins (17, 18, 50). Notably, the function of SLAMF7 and the identification of its adaptors involved in cytokine modulation in other cell types have not been explored. Our findings showed that the phosphorylation of SHIP1 decreased in SLAMF7-KO macrophages and that the proinflammatory cytokines downregulated by SLAMF7 were reversed by knockdown of SHIP1. Furthermore, we found that SLAMF7 directly interacted with SHIP1 in murine macrophages. Supporting our findings, a previous study showed that SHIP1 and SLAMF7 interact in MM cells depending on Src kinase and that elotuzumab triggers the tyrosine phosphorylation of SHIP1 (18). However, we failed to detect the alteration of proinflammatory cytokines in SLAMF7-overexpressed cells with LPS stimulation after Src and Syk kinase inhibition. In addition, the NF-κB–related kinase TBK1 was found to participate in SLAMF7-regulated cytokine production.

SHIP1 is a potent endogenous inhibitor of the NF-κΒ signaling pathway (75, 76). SHIP1 possesses an amino-terminal SH2 domain that binds preferentially to the sequence pY (Y/S/T) L (M/L), a centrally located EEP domain that selectively hydrolyzes the 5′-phosphate and a P-rich domain that binds a subset of SH3-containing proteins (48). Although SHIP1-mediated inhibition of TLR4-induced PI3K activation is dependent on its phosphatase activity (77), An et al. found that phosphatase activity–disrupted mutant SHIP1 (with mutation of the P671, D675, and R676 sites in the phosphatase activity domain) remains inhibitory to LPS-induced TNF production (77). Here, we demonstrated that the phosphatase domain of SHIP1 mediated the interaction with SLAMF7 and assisted in inhibiting the autoubiquitination of TRAF6. It has been reported that SLAMF7 has a unique cytoplasmic domain distinct from other SLAMF members, which is not the typical binding sequence for SAP (78). Our data showed that the interaction between SLAMF7 and SHIP1 was impaired after KO of the EEP domain but not the SH2 or P-rich domain of SHIP1. Thus, we supposed that the EEP domain of SHIP1 has an essential role in the interaction between SLAMF7 and SHIP1 upon activation of TLR signaling. In addition, we showed that a lack of any one of the functional domains of SLAMF7 rendered it unable to interact with SHIP1, indicating the indispensability of full-length SLAMF7 in SLAMF7-SHIP1 interactions. As we discussed above, there are 3 critical tyrosine phosphorylation sites within SLAMF7 (49, 50). Guo et al. reported that the long transcript of SLAMF7 (SLAMF7-L) in MM cells, which contains all tyrosines, binds to SHIP1 rather than the short transcript of SLAMF7 (SLAMF7-S) (46), which bears an alternative intracytoplasmic domain lacking Y261. However, we discovered that the Y281 mutation, but not the Y261 and Y281 mutations, abolished the interaction between SLAMF7 and SHIP1, although these tyrosines are reported to couple with SAP or EAT-2 in NK cells (15, 17).

Ubiquitination modification of signaling proteins through K48-linked ubiquitin chains typically leads to protein degradation by proteasome-dependent mechanisms. However, ubiquitination modification through non-K48 linkages such as K63 ubiquitin linkages can activate multiple signaling pathways like NF-κB (43). As a E3 ubiquitin ligase, TRAF6 participates in the inflammatory response via the regulation of immune pathways (79–82). Recently, a large number of negative regulators have been reported to suppress the autoubiquitination of TRAF6, in which the deubiquitinating enzyme A20 is required for the termination of TLR activity by removing ubiquitin from TRAF6 (80). Moreover, the inhibition of NF-κB activation by cylindromatosis (CYLD) is mediated by the deubiquitination and inactivation of TRAF2 and TRAF6, which downregulate the secretion of cytokines (79). The heat shock protein HSP70 inhibits LPS-induced NF-κB activation by binding with TRAF6 and preventing its ubiquitination (81). Kim et al. discovered that inositol polyphosphate multikinase (IPMK) noncatalytically enhances TLR signaling by stabilizing TRAF6 in macrophages and that IPMK depletion blunts TLR-mediated signaling and proinflammatory cytokine secretion, thus rendering mice resistant to septic responses (82). Our results showed that SLAMF7 inhibited TRAF6 K63 ubiquitination by cooperating with SHIP1 and interacted with TRAF6 independent of 3 tyrosine sites. Knockdown of SLAMF7 increased the overall protein ubiquitination in BMDMs independent of the proteasome pathway, suggesting that SLAMF7 had no influence on the proteasome degradation. Li and colleagues found that arrestin 2 associates with p-TIGIT for further recruitment of SHIP1 to impair TRAF6 autoubiquitination, leading to the suppression of IFN-γ production in NK cells. Here, we demonstrated that the EEP domain of SHIP1 facilitated the inhibition of TRAF6 ubiquitination. SHIP1 contains 3 functional domains, including an SH2 domain, a P-rich domain, and an EEP domain. As for the interaction of SHIP1 with other molecules, Bao and colleagues reported that the adaptor CD2AP forms a complex with SHIP1 by binding to the P-rich domain of SHIP1, with the first SH3 domain beimg associated with Cbl in plasmacytoid DCs (47). As shown in our results, SHIP1 interacted with SLAMF7 via the EEP domain, and the P-rich domain was available for interaction with other molecules. Collectively, we demonstrated that SLAMF7 recruited SHIP1 and TRAF6 to inhibit proinflammatory cytokine production.

In summary, this study provides evidence that SLAMF7 is an important negative regulator of sepsis-induced inflammation and reveals that SLAMF7 cooperates with SHIP1 to inhibit TRAF6 ubiquitination and suppress proinflammatory cytokine production. Our study sheds light on the regulatory role of SLAMF7 in sepsis and uncovers the interaction between SLAMF7 and TRAF6/SHIP1 to transmit downstream signals, which may provide support for the development of therapeutic strategies for sepsis.

## Methods

### Study participants.

Patients were diagnosed as having sepsis according to the guidelines from The Third International Consensus Definitions for Sepsis and Septic Shock (Sepsis-3) (83). Patients with sepsis were included in the study if they met the following criteria: (a) a clear indication of infection; (b) secondary organ dysfunction or acute exacerbation of primary organ dysfunction; and (c) a Sequential Organ Failure Assessment (SOFA) score of 2 or higher. The clinical characteristics of the patients were noted upon their enrollment in the study (Supplemental Tables 1 and 2). A total of 81 age-matched healthy controls with no history or clinical disease were enrolled.

### Mice.

WT C57BL/6 mice were purchased from the Sun Yet-sen University Animal Supply Center. *SLAMF7*-KO mice and SLAMF7^fl/fl^ mice (mice with loxP-flanked alleles of SLAMF7 exons 3, 4, 5, and 6) were generated at GemPharmatech. *TLR4*-KO mice and Lyz2^Cre^ mice (expressing Cre recombinase under the control of a Lyz2 promoter) were purchased from GemPharmatech. The targeting strategy was used to generate *SLAMF7*-KO mice with a complete deletion of exons 3, 4, 5, and 6 of the *SLAMF7* gene. Mice were backcrossed with mice on the C57BL/6J background for more than 6 generations. All mice were genotyped by PCR using genomic DNA obtained from tail biopsies. All animals were genotyped for the SLAMF7 mutation (Supplemental Figure 15A). Flow cytometry was performed to confirm the absence SLAMF7 protein, as indicated by negative SLAMF7 expression on splenic CD11b^+^cells from *SLAMF7*-KO mice (Supplemental Figure 15B). To generate mice with myeloid-specific KO of the SLAMF7 allele, SLAMF7^fl/fl^ mice were crossed with Lyz2^Cre^ mice to achieve Lyz2-specific deletion of SLAMF7 (SLAMF7^fl/fl^ Lyz2^Cre^) (Supplemental Figure 15C). Six- to 8-week-old male mice were used in the experiments.

### Establishment of the sepsis model.

The endotoxin shock mouse model was established by injecting mice i.p. with LPS (L2880, MilliporeSigma) (nonlethal: 20 mg/kg; lethal: 40 mg/kg). For bacteria-induced sepsis, mice were intratracheally infected with *P*. *aeruginosa* at 5 × 10^8^ CFU/kg body weight. Polymicrobial sepsis was induced using the CLP method as described previously (51). Brieﬂy, mice were anesthetized with injection of 0.4 g/kg chloral hydrate. A small midline incision was made to expose the cecum through the skin and peritoneum under aseptic conditions. Approximately 75% of the cecal appendage was ligated midway between the cecal base and the distal pole, using 4/0 surgical silk. Then, double punctures were made using an 18 gauge needle, expelling a small amount of feces into the abdominal cavity. The cecum was then returned to the peritoneal cavity, and the incision was closed using 2 layers of sutures. Sham-operated mice were subjected to the same procedures without cecal puncture. For SLAMF7 activation in vivo, rmSLAMF7 protein (100 μg/kg) (4628-SF, R&D Systems) was injected i.p. 6 hours before or after sepsis induction.

### Establishment of the sepsis-induced immunosuppression model.

Polymicrobial sepsis was induced using the CLP method. In brief, cecal ligation and puncture were performed in mice with an 18 or 23 gauge needle to establish lethal or moderate CLP sepsis, respectively. For antibiotic treatment, mice were given an i.p. injection of ertapenem sodium (30 mg/kg, Merck) beginning 6 hours after CLP and continuing every 12 hours for the first 3 days. After 15 days, mice that survived sepsis were infected with 2 × 10^7^ CFU *P*. *aeruginosa* per kilogram body weight.

### Macrophage depletion.

Liposomes, composed of phospholipid bilayers and containing dichloromethylene diphosphonate (clodronate liposomes), or PBS (control liposomes) were purchased from Liposoma BV. A total of 100 μL clodronate-containing liposome suspension was injected i.p. into WT and *SLAMF7*-KO mice as previously described (52). Macrophages were depleted in the peritoneal lavage for up to 1 week after clodronate liposomes injection.

### Plasmid construction and transient transfection.

The cDNA sequences of murine TRAF6, SLAMF7, and SHIP1 with an N-terminal tag were amplified by reverse transcription PCR and subcloned into the pcDNA3.1(+) vector following the manufacturer’s protocol (GenScript). The SLAMF7-Δextra, SLAMF7-Δtrans, and SLAMF7-Δintra were generated by deleting the extracellular domain, the transmembrane domain, or the intracellular domain. The tyrosine mutations of SLAMF7 at Y261, Y266, and Y281 were identified as previously described (19) and induced using a MultiS Fast Mutagenesis kit V2 (Vazyme). The SH2, EEP, and P-rich domains of SHIP1 were generated from a full-length SHIP1 plasmid. The SHIP1-ΔSH2, SHIP1-ΔEEP, and SHIP1-ΔP- rich plasmids were generated by deleting key domains, as described above. All clones were sequenced and expression was confirmed by immunoblotting transiently transfected HEK 293T (American Type Culture Collection [ATCC], CRL-11268) cell lysates with anti-Tag antibodies. RAW264.7 cells were transiently transfected with plasmids or blank vector using Lipofectamine 2000 (Invitrogen, Thermo Fisher Scientific) as a previously described (23). All siRNAs used were purchased from RiboBio. The negative control siRNA sequence (siN05815122147) was supplied by RiboBio. The siRNA target sequences are listed in Supplemental Table 3.

### Statistics.

Statistical analysis was performed using GraphPad Prism 7 (GraphPad Software), and data are shown as the mean ± SEM, unless otherwise stated. For 2-group comparisons, a 2-tailed, unpaired Student’s *t* test was used. A 2-way, nonparametric ANOVA was used followed by Tukey’s multiple-comparison test for multigroup comparisons. All FACS analysis, immunofluorescence analysis, and Western blots were repeated in at least in 3 independent experiments. A *P* value of less than 0.05 was considered statistically significant.

### Study approval.

All experimental protocols were approved by Sun Yat-sen University. The methods used in this study were carried out in accordance with the approved guidelines. All animal experiments were performed in accordance with the NIH’s *Guide for the Care and Use of Laboratory Animals* (National Academies Press, 2011), with the approval of the scientific investigation board and animal ethics committee of The Fifth Affiliated Hospital of Sun Yat-sen University (Guangdong, China). The human cohort study was approved by the IRB of The Fifth Affiliated Hospital of Sun Yat-sen University. All participants provided written informed consent for their participation in the study.

## Author contributions

YW, JL, ML, QW, HT, and SM conducted the experiments and acquired data. MW, L Liu, SG, L Li, and XH provided scientific expertise and reagents. YW, JL, ML, SM, and XH designed the study, interpreted the data, and wrote the manuscript. All authors read the final version of the manuscript and approved the submission.

## Supplementary Material

Supplemental data

## Version 1 02/07/2023

In-Press Preview

## Version 2 03/15/2023

Electronic publication

## Version 3 10/30/2026

Corrected SLAMF7 (human) quantitative primers in Supplemental Table 4
