# Corrigendum to SLAMF7 regulates the inflammatory response in macrophages during polymicrobial sepsis

**DOI:** 10.1172/JCI213718

**Published:** 2026-11-02

**Authors:** Yongjian Wu, Qiaohua Wang, Miao Li, Juanfeng Lao, Huishu Tang, Siqi Ming, Minhao Wu, Sitang Gong, Linhai Li, Lei Liu, Xi Huang

Original citation: *J Clin Invest*. 2023;133(6):e150224. https://doi.org/10.1172/JCI150224

Citation for this corrigendum: *J Clin Invest*. 2026;136(21):e213718. https://doi.org/10.1172/JCI213718

The authors recently became aware that in Supplemental Table 4, the SLAMF7 (human) quantitative primers were incorrectly noted. The correct sequences are provided in the updated supplemental PDF.

The authors regret the error.

## Version 1 11/02/2026

Electronic publication

