# Supplementary material for: SLAMF7 regulates the inflammatory response in macrophages during polymicrobial sepsis
Source: J Clin Invest. 2023 Mar 15;133(6):e150224. doi: 10.1172/JCI150224 (PMC10014109; doi:10.1172/JCI150224)
Supplement: Supplemental data [file jci-133-150224-s133.pdf]

SLAMF7 Regulates Inflammatory Response in Macrophages During Polymicrobial Sepsis

Yongjian Wu[1], Qiaohua Wang[1], Miao Li[2], Juanfeng Lao[1], Huishu Tang[1], Siqi Ming[3], Minhao Wu[1], Sitang Gong[4], Linhai Li[5], Lei Liu[3] and Xi Huang[1, 3, 5, *]

[1] Center for Infection and Immunity and Guangdong Provincial Key Laboratory of Biomedical Imaging, the Fifth Affiliated Hospital of Sun Yat-sen University, Zhuhai, Guangdong Province, 519000, China.

[2] Scientific Research Center, The Seventh Affiliated Hospital, Sun Yat-sen University, Shenzhen, Guangdong Province, 518107, China.

[3] National Clinical Research Center for Infectious Disease, Shenzhen Third People's Hospital; the Second Affiliated Hospital of Southern University of Science and Technology, Shenzhen, Guangdong Province, 518112, China.

[4] Department of Gastroenterology, Guangzhou Women and Children's Medical Center, Guangzhou Institute of Pediatrics, Guangzhou Medical University, Guangzhou, Guangdong Province, 510623, China.

[5] The Sixth Affiliated Hospital of Guangzhou Medical University, Qingyuan People's Hospital, Qingyuan, Guangdong Province, 511518, China.

* Corresponding author

Supplemental Figures

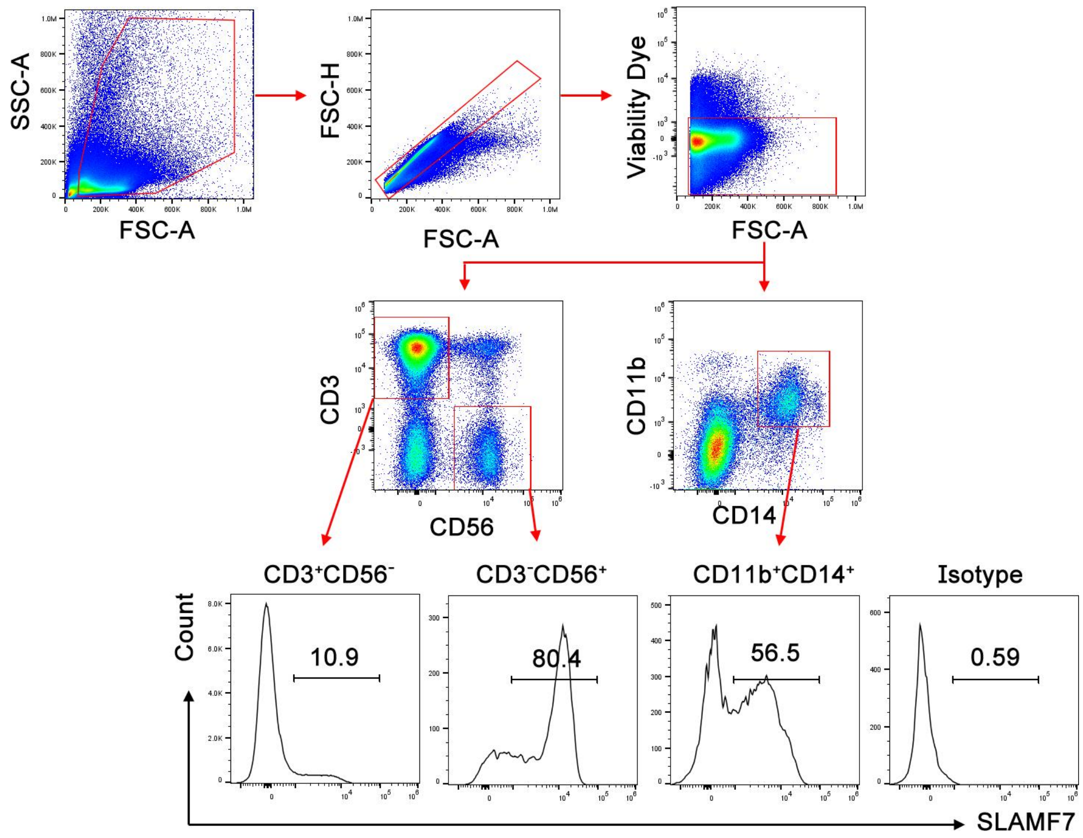

Supplemental Figure 1. Gate strategy. Cells were gated as single, live (Viability dye^{-}) cells. T cells, NK cells and monocytes were gated as CD3^{+}CD56^{-}, CD3^{-}CD56^{+} and CD11b^{+}CD14^{+} cells, respectively. SLAMF7 Ab and IgG isotype were used to set the cutoff value of SLAMF7^{+} cells and SLAMF7^{-} cells.

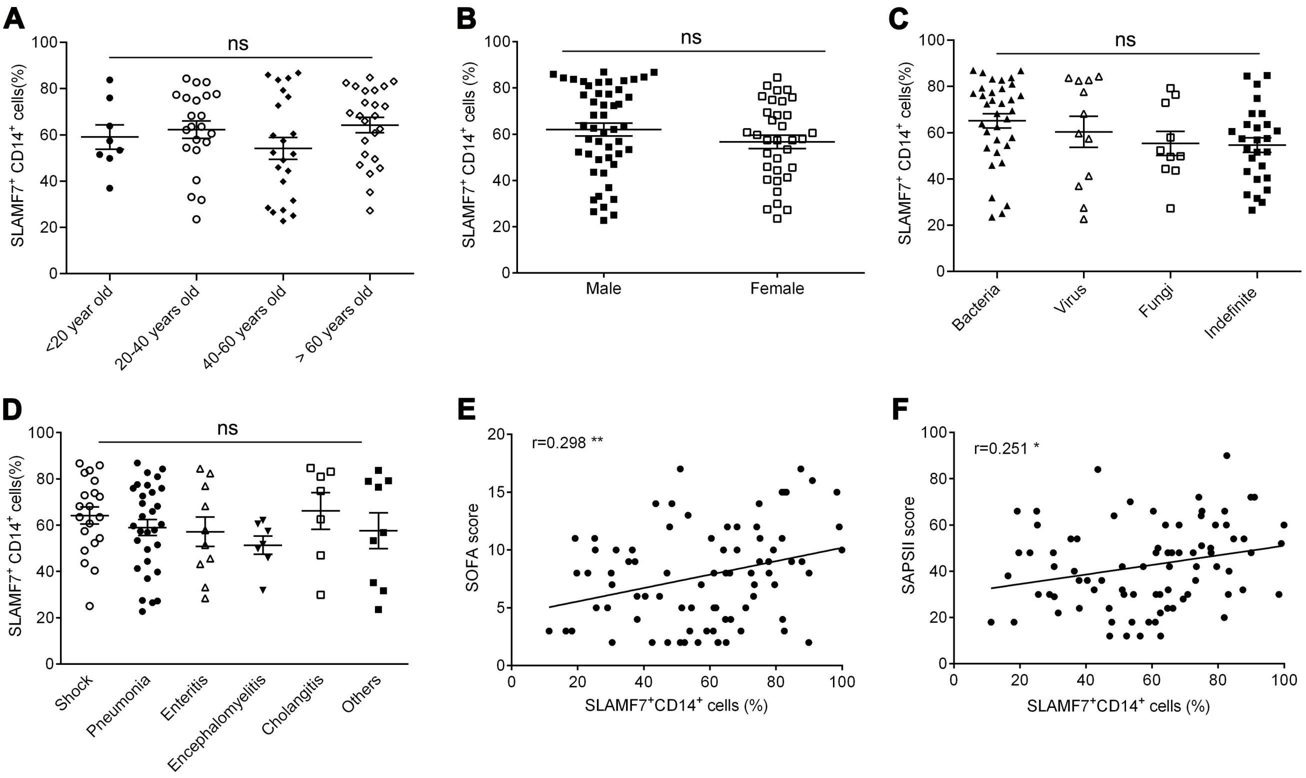

Supplemental Figure 2. Classified expression of SLAMF7 and the correlation between SLAMF7 expression and disease severity parameter. Percentage of SLAMF7^{+} CD11b^{+} cells in PBMC from sepsis patients were divided by (**A**) gender, (**B**) age, (**C**) infectious microbes or (**D**) diagnosis. (**E** and **F**) The correlations of the percentage of SLAMF7^{+} monocytes with SOFA **(E)** and SAPSII **(F)** score in sepsis patients. ns, not significant, **P*< 0.05, and ***P*< 0.01, by One way ANOVA (**A-D**) and Spearman correlation (**E** and **F**).

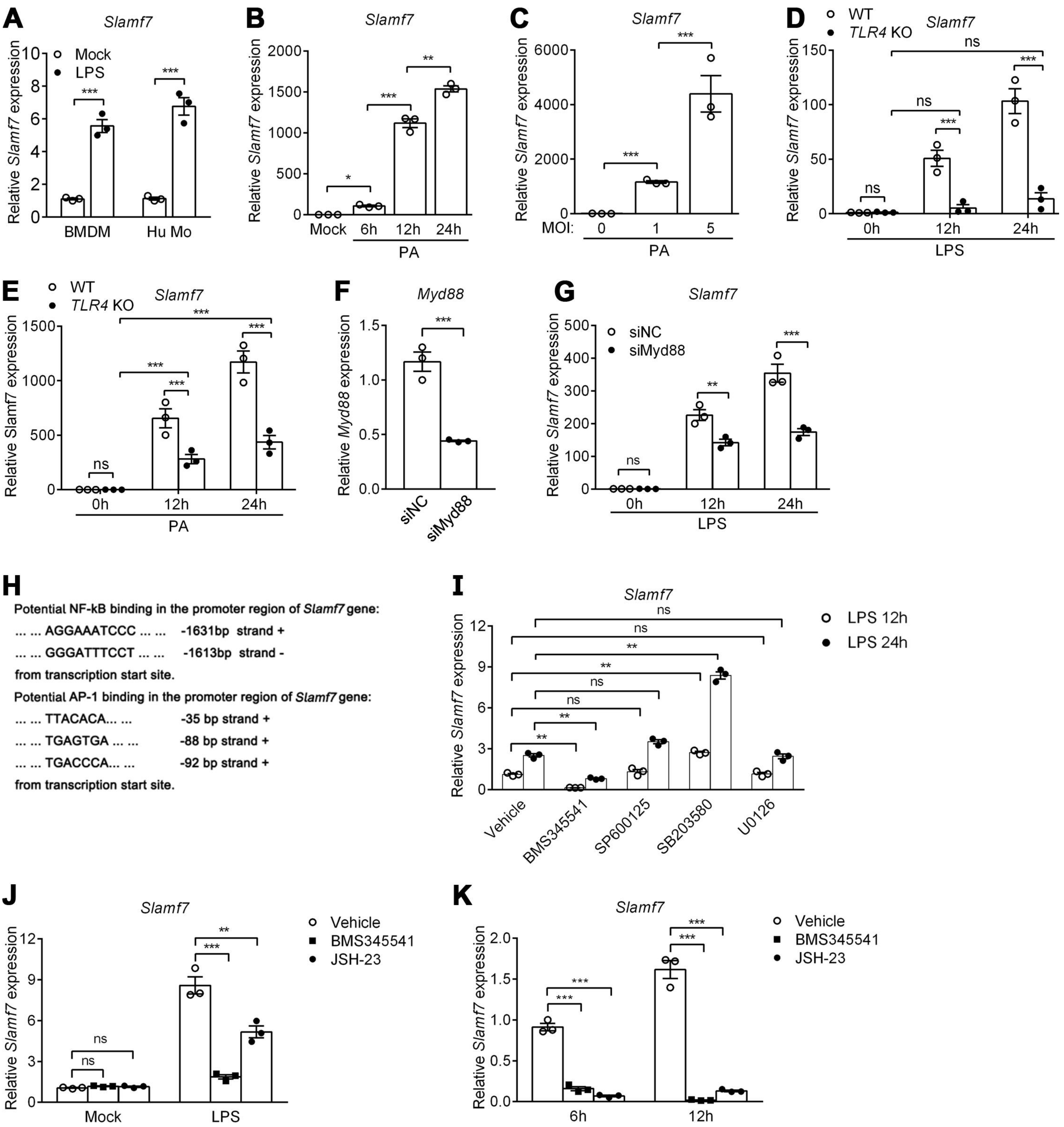

Supplemental Figure 3. TLR/NF-κB signaling induces SLAMF7 expression in macrophages.

(**A**) mRNA levels of *Slamf7* in mouse bone marrow derived macrophages (BMDM) or human monocytes isolated from peripheral blood (monocytes) were examined after LPS stimulation for 24 hours. (**B** and **C**) mRNA levels of *Slamf7* were examined in RAW264.7 cells after PA infection (MOI=1) at indicated time points (**B**) or indicated concentrations at 24 hours post PA stimulation (**C**). (**D** and **E**) mRNA levels of *Slamf7* were examined in WT vs *TLR4* KO BMDMs after LPS treatment

(**D**) or PA infection (**E**) at indicated time points. (**F** and **G**) BMDM were transiently transfected with MyD88 specific or control siRNA for 24h, followed by LPS stimulation for 24 hours. mRNA levels of *Myd88* (**F**) and *Slamf7* (**G**) were examined by real-time PCR. (**H**) The potential binding sites for transcription factors including NF-κB (-1631bp from the transcription start site) and AP-1 (-35bp, -88bp and -92bp from the transcription start site) were analyzed in the promoter region of *Slamf7* by JASPAR software. (**I**) *Slamf7* expression was examined in RAW264.7 cells pre-treated with inhibitors targeting IKKα/β (BMS345541), JNK (SP600125) or p38 (U0126) for 1 hours, followed by LPS challenge for 24 hours. (**J** and **K**) *Slamf7* expression in BMDMs (**J**) or RAW264.7 cells (**K**) after pretreatment with NF-κB signaling inhibitors (BMS345541 for IKKα/β and JSH-23 for NF-κB) and LPS stimulation. Data represent the mean ± SEM from at least 3 independent experiments. ns, not significant. * $P < 0.05$, ** $P < 0.01$, and *** $P < 0.001$, by unpaired Student's t test (**A**, **F** and **G**) and one way ANOVA (**B-E** and **I-K**).

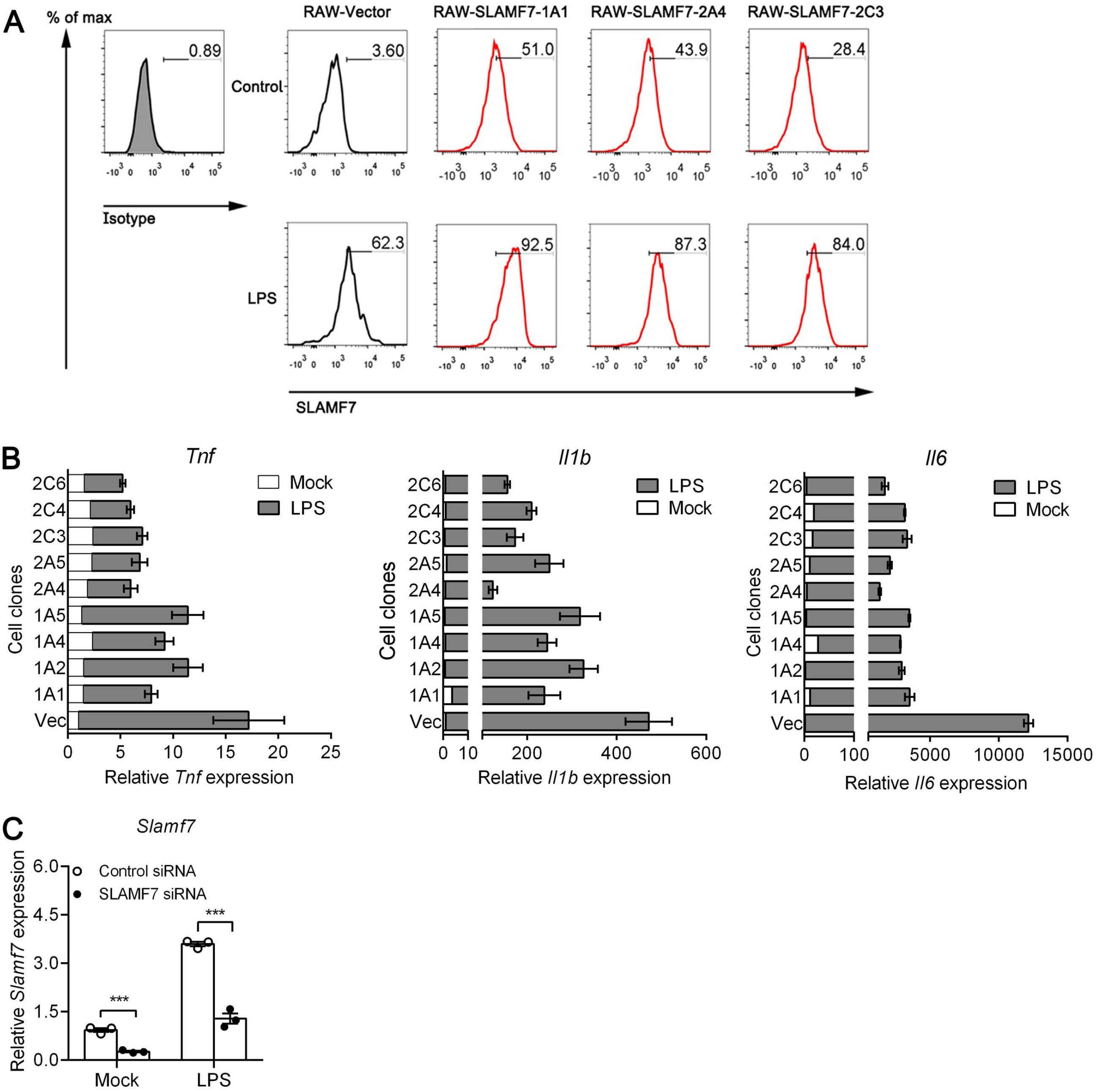

Supplementary Figure 4. SLAMF7 negatively regulates inflammatory cytokines production *in vitro*. (**A**) SLAMF7 expression in RAW-Vector cells and multiple RAW-SLAMF7 overexpressing cell clones before and after LPS stimulation for 24 hours. (**B**) RAW264.7 cells stably expressing SLAMF7 (RAW-SLAMF7) and control vector (RAW-Vector) cells were constructed. mRNA levels of inflammatory cytokines in different RAW-SLAMF7 cell clones vs RAW-Vector cells were analyzed. (**C**) The expression of *Slamf7* in BMDM transfected with SLAMF7 siRNA or Control siRNA, followed by LPS stimulation for 24 hours. Data represent the mean ± SEM from at least 3 independent experiments. *** P <0.001, by unpaired Student's t test (**C**).

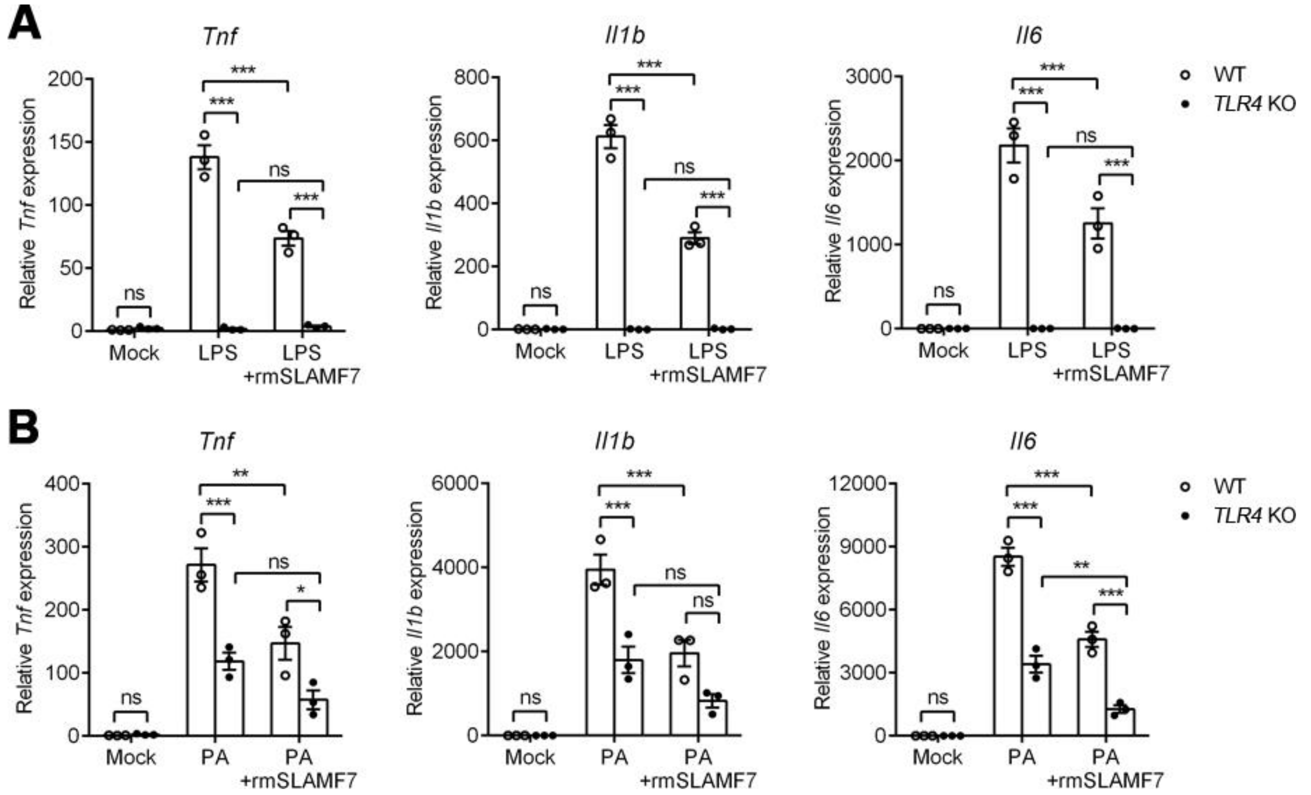

Supplementary Figure 5. SLAMF7 regulated pro-inflammatory cytokines in TLR4 dependent and independent manner. (**A**) mRNA levels of *Tnf, Il1b* and *Il6* were examined in BMDMs isolated from WT or TLR4 KO mice after LPS stimulation for 24 hours. (**B**) mRNA levels of *Tnf, Il1b* and *Il6* were examined in WT or TLR4 KO BMDMs after PA infection for 24 hours. Data represent the mean ± SEM from at least 3 independent experiments. ns, not significant. * $P < 0.05$, ** $P < 0.01$, and *** $P < 0.001$, by Two-way ANOVA test (**A** and **B**).

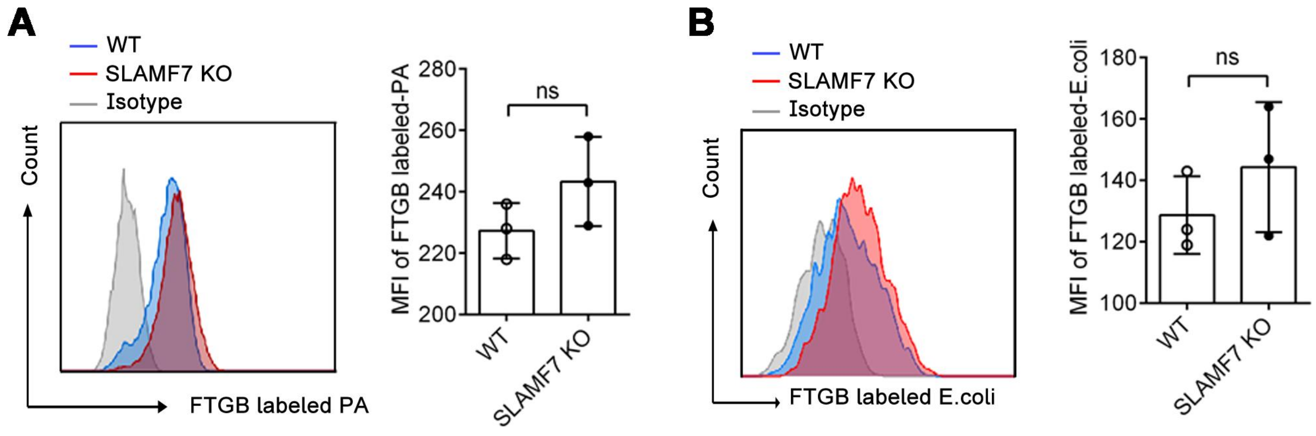

Supplementary Figure 6. SLAMF7 did not affect bacterial phagocytosis of macrophage. WT or SLAMF7 KO BMDM were infected with FTGB labeled PA (**A**) or SA (**B**), and then the efficiency of phagocytosis was determined by flow cytometry. ns, not significant, by unpaired Student's t test (**A and B**).

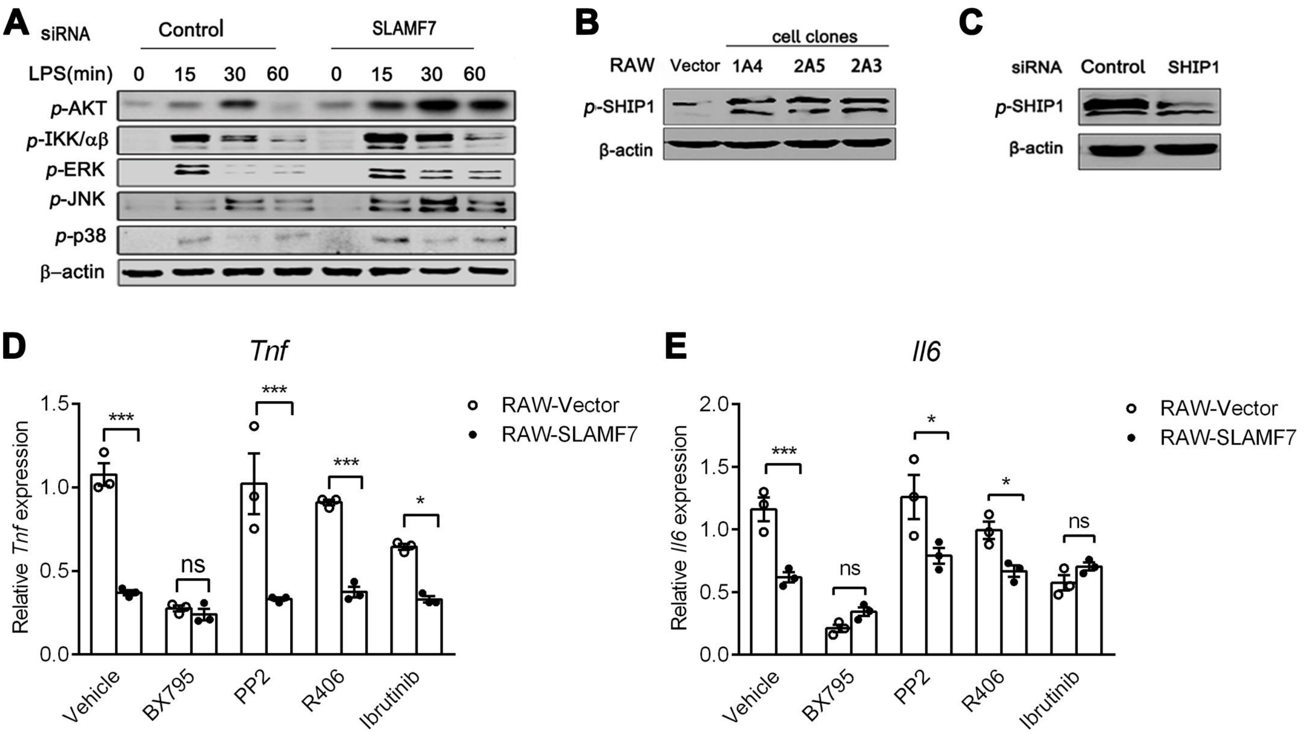

Supplementary Figure 7. SLAMF7 inhibits MAPKs/NF-kB signaling pathways requiring TBK1 kinases. (**A**) Immunoblot analysis of phosphorylated signaling molecules in MAPKs and AKT signaling pathways of RAW264.7 cells transfected with SLAMF7 siRNA and Control siRNA after stimulating for 0, 15, 30 and 60 minutes. (**B** and **C)** Phosphorylation of SHIP1 in RAW-SLAMF7 cell clones compared to RAW-Vector (**B**) or after transfected with siRNA targeting SHIP1 (**C**). (**D** and **E)** The expression of *Tnf* (**D**) and *Il6* (**E**) of RAW264.7 cells after pre-transfected with inhibitors of TBK1 (BX795), Src (PP2), Syk (R406) and Btk (Ibrutinib), followed by LPS stimulation for 6 hours. Data represent the mean ± SEM from at least 3 independent experiments. ns, not significant. * $P < 0.05$ and *** $P < 0.001$, by unpaired Student's t test (**D** and **E**).

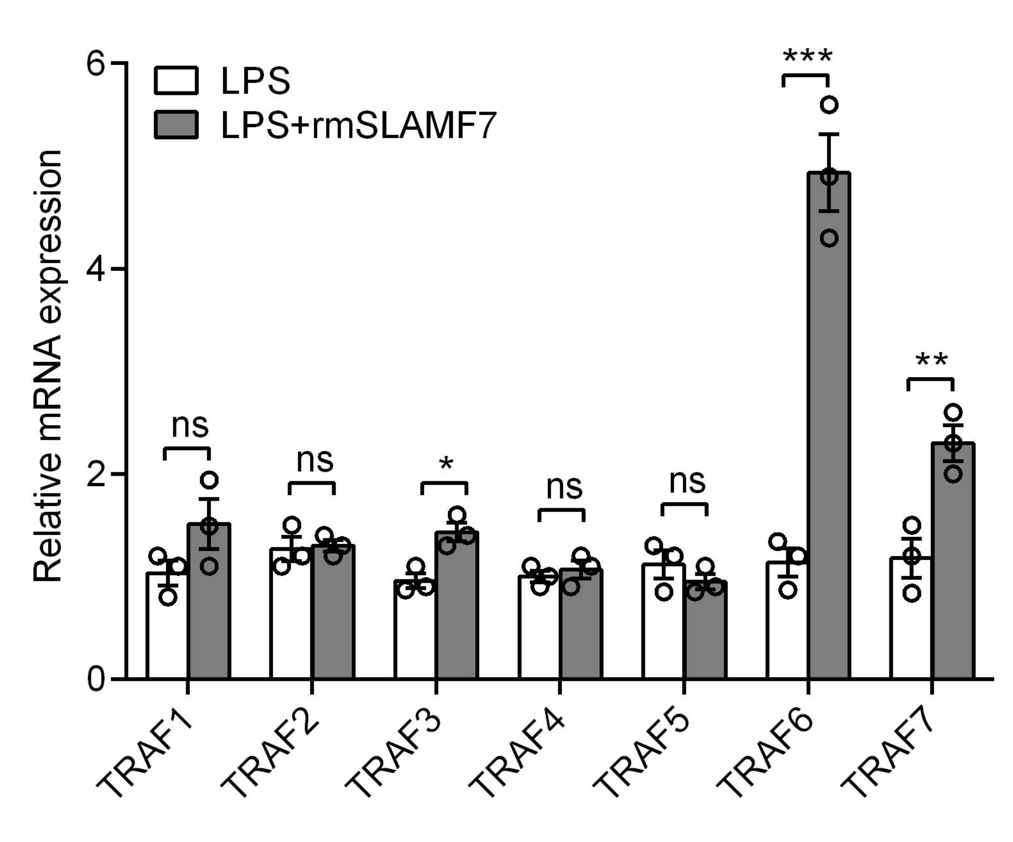

Supplementary Figure 8. The expression levels of *TRAF1-7* in BMDM after treatment with rmSLAMF7, followed by LPS stimulation. Data represent the mean ± SEM from at least 3 independent experiments. ns, not significant. * $P < 0.05$, ** $P < 0.01$, and *** $P < 0.001$, by unpaired Student's t test.

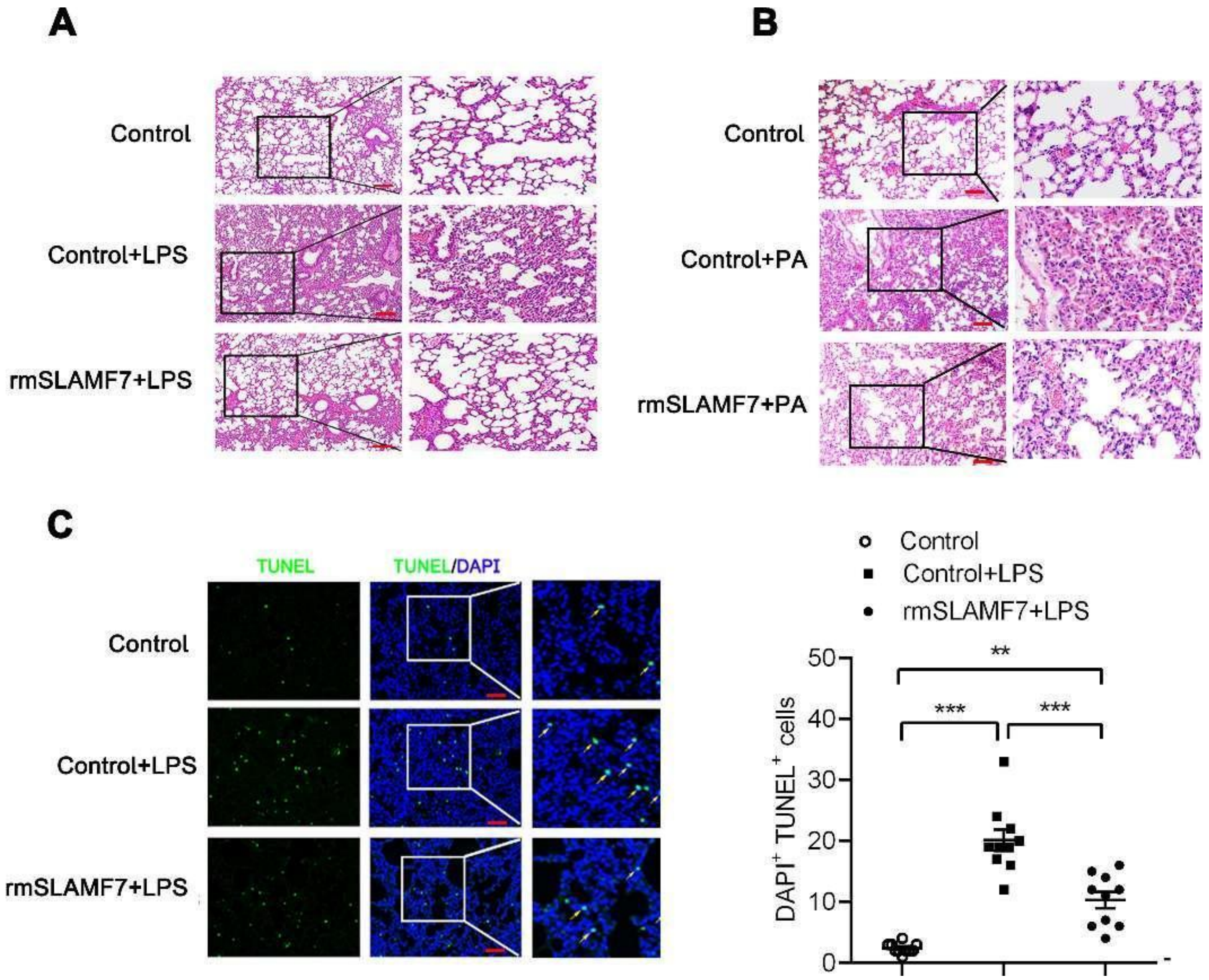

Supplementary Figure 9. Activating SLAMF7 attenuated lung injury in septic mice. (**A** and **B**) Sections from mice challenged with LPS (25mg/kg) (**A**) or PA (2x10^7 CFU/kg) (**B**) for 1 hours, followed by the intraperitoneal injection of rmSLAMF7 or Control (0.9% NaCl) for 6 hours. (**C**) The TUNEL and DAPI co-localization cells of lung section from endotoxin mice. White square showed the area to zoom in. Yellow arrows point the co-localization of DAPI and TUNEL. Scale bars, 20 μm. Data represent the mean ± SEM from at least 3 independent experiments. ** $P < 0.01$ and *** $P < 0.001$, by one way ANOVA (**C**).

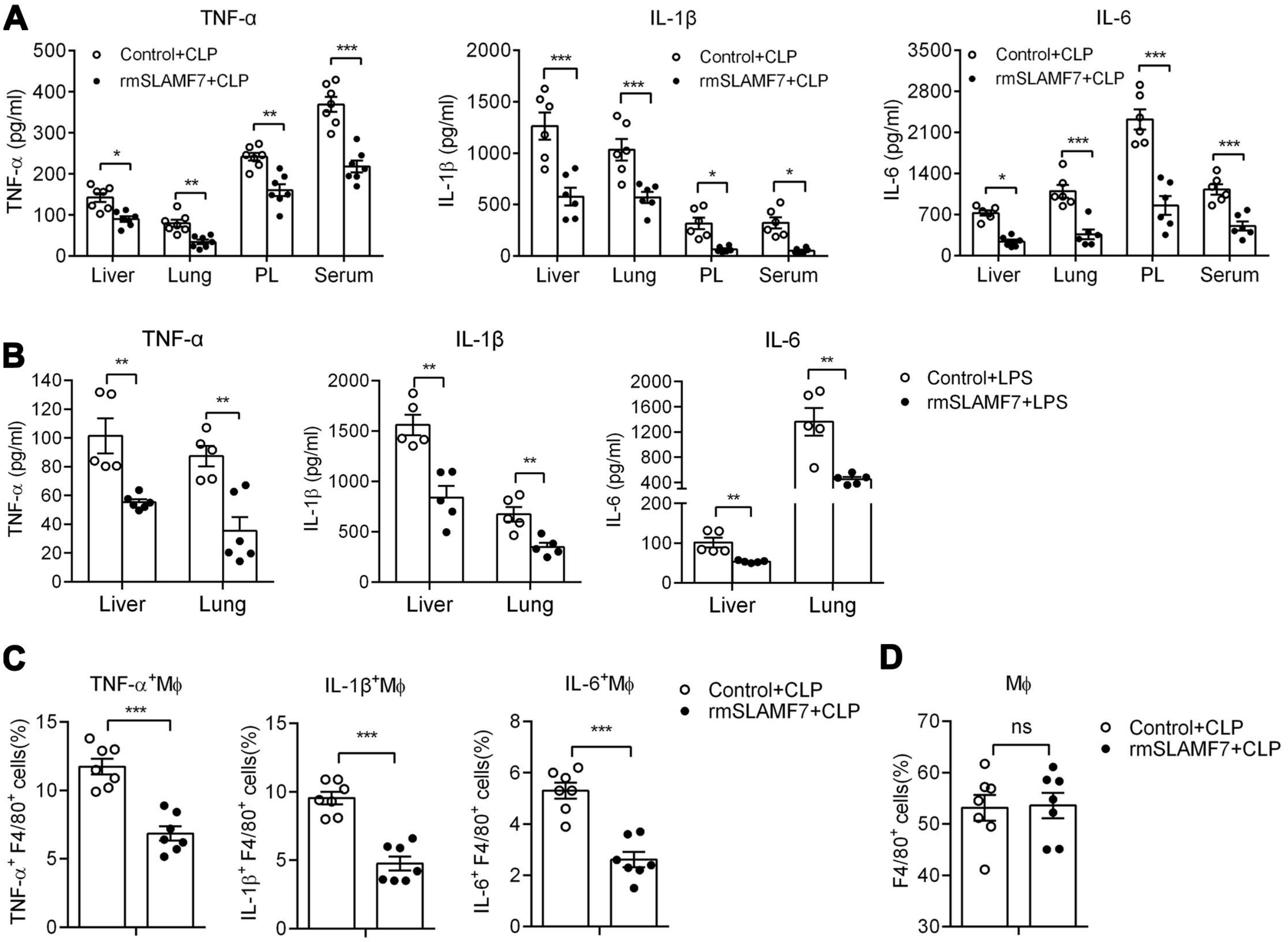

Supplementary Figure 10. SLAMF7 activation reduced inflammatory cytokines production *in vivo*. A-D, Mice were intraperitoneally (i.p.) injected with rmSLAMF7 or vehicle control (0.9% NaCl), followed by the establishment of LPS (25mg/kg) or CLP sepsis model 6 hours later. (**A and B**) The levels of TNF-α, IL-1β and IL-6 in the supernatant of liver, lung or peritoneal lavage (PL) and the serum were detected by ELISA 6 hour post CLP (**A**) or LPS (**B**) challenge. (**C** and **D)** The percentage of TNF-α^+, IL-1β^+ or IL-6^+F4/80^+ cells (**C**) and the percentage of F4/80^+ macrophage in PL were respectively determined by flow cytometry in CLP sepsis model 6 hours later. Data represent the mean ± SEM from at least 3 independent experiments. ns, not significant. * $P < 0.05$, ** $P < 0.01$, and *** $P < 0.001$, by unpaired Student's t test (**A**-**D**).

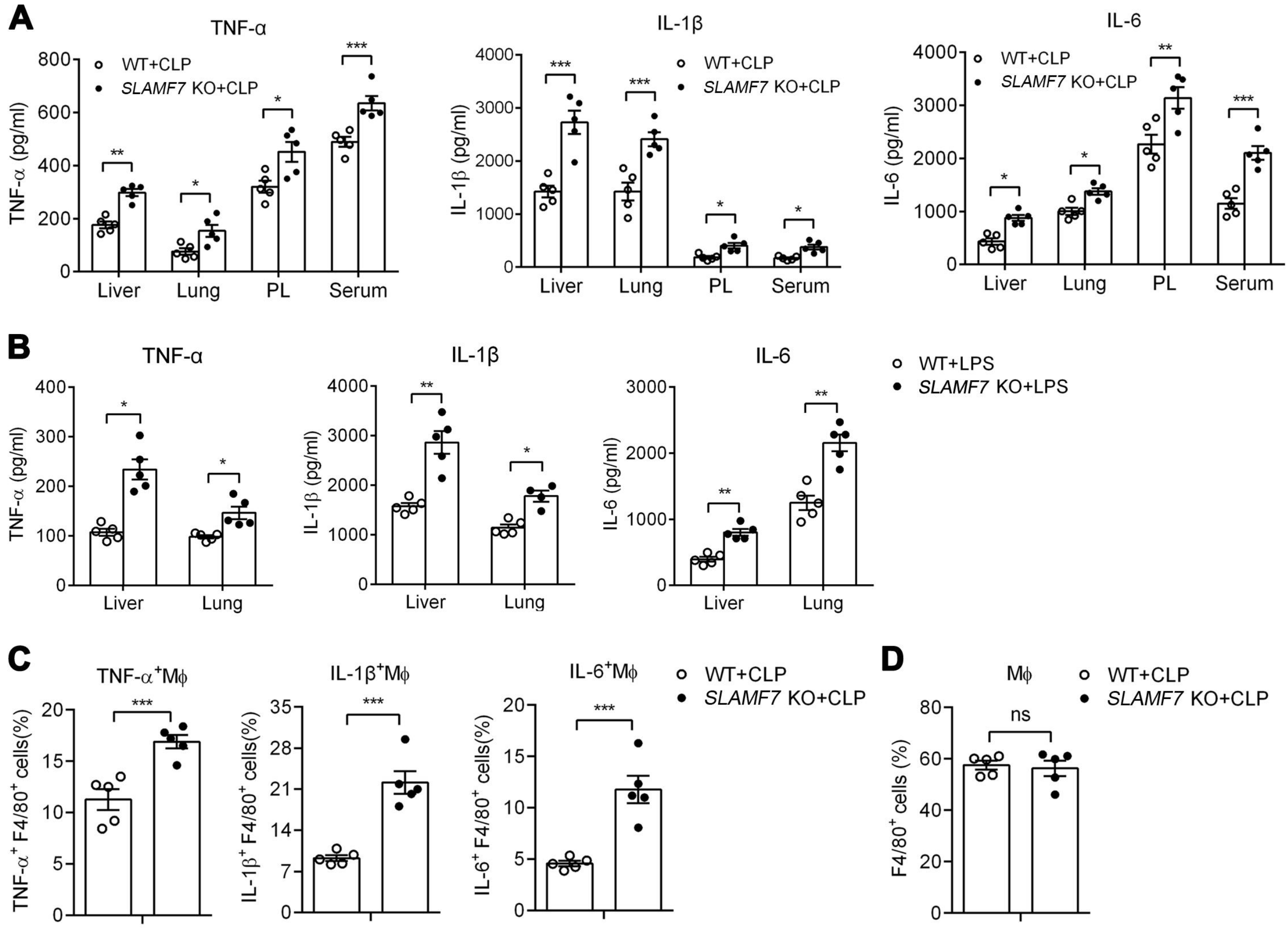

Supplementary Figure 11. SLAMF7 deficiency up-regulated inflammatory cytokines production *in vivo.* (**A** and **B**) WT and *SLAMF7* KO mice were performed with CLP surgery (**A**) or i.p. injected with LPS (25mg/kg) (**B**). The levels of TNF-α, IL-1β and IL-6 in the supernatant of liver, lung or PL and serum were measured by ELISA 6 hours later. (**C** and **D**) CLP sepsis model was established in WT and *SLAMF7* KO mice. The percentage of TNF-α^+, IL-1β^+ or IL-6^+ F4/80^+ cells (**C**) and the percentage of F4/80^+ macrophage (**D**) in PL were analyzed by flow cytometry at 6 hours after CLP. Data represent the mean ± SEM from at least 3 independent experiments. ns, not significant. * $P < 0.05$, ** $P < 0.01$, and *** $P < 0.001$, by unpaired Student's t test (**A-D**).

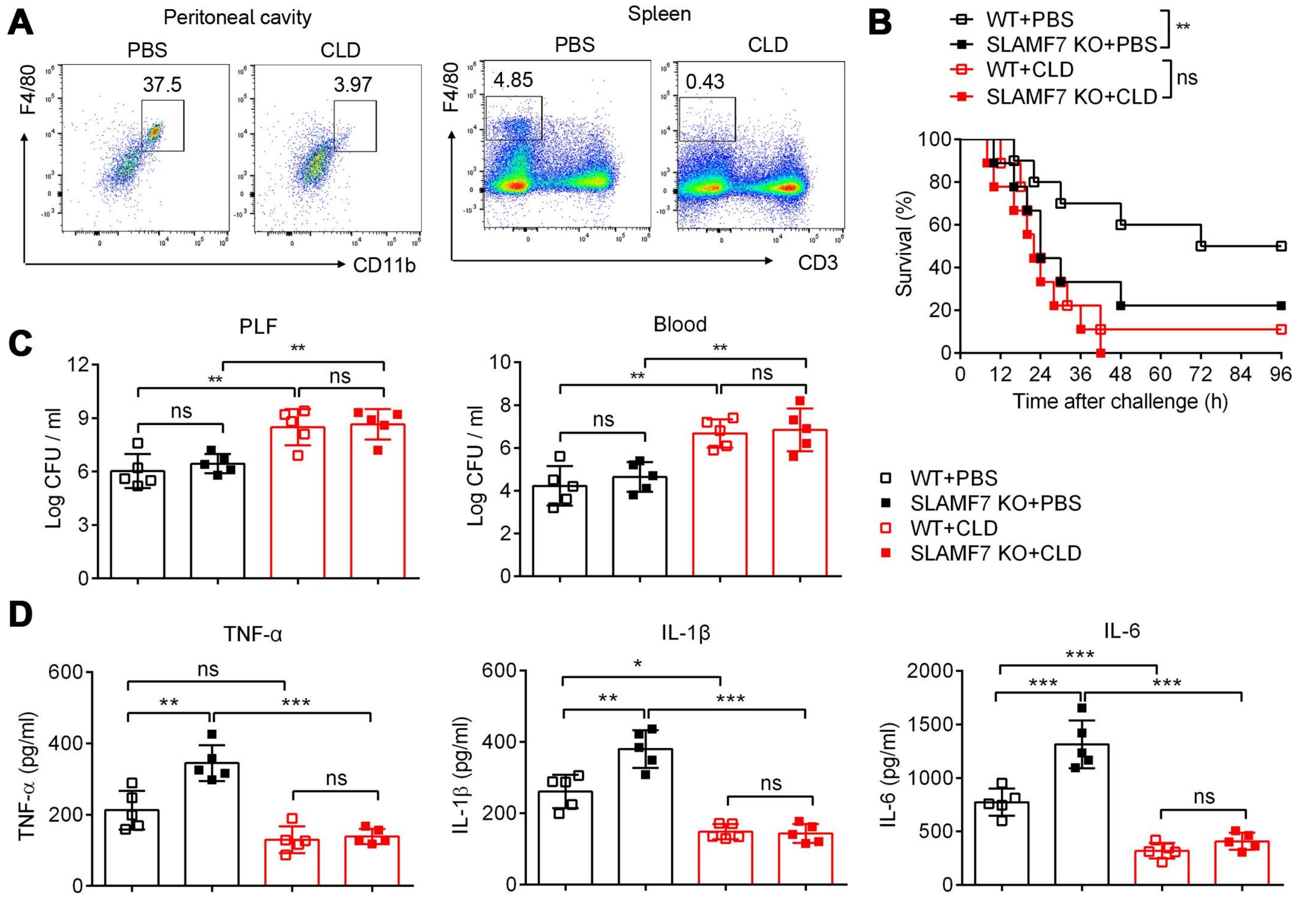

Supplementary Figure 12. Macrophage is the major subset in SLAMF7-mediated effects in sepsis. WT and *SLAMF7* KO mice were injected with clodronate liposomes (CLD) or control liposomes (PBS) for one week, followed by the establishment of CLP polymicrobial sepsis model. (**A**) Clearance efficiency of macrophage was determined in WT mice by detecting the percentage of macrophage in the peritoneal cavity (Gated as $CD11b^{+}F4/80^{+}$ cells) and spleen (Gated as $CD3^{-}F4/80^{+}$ cells) one week after the injection of CLD. (**B**) Survival rate was observed. (**C**) Bacterial loads in blood and peritoneal lavage of CLP mice were quantified by colony forming units (CFU) assay 24 hour later. (**D**) The concentrations of TNF-α, IL-1β and IL-6 in the serum were determined by ELISA. Data represent the mean ± SEM from at least 3 independent experiments. ns, not significant. * $P < 0.05$, ** $P < 0.01$, and *** $P < 0.001$, by Log-rank test (**B**) and one way ANOVA (**C** and **D**).

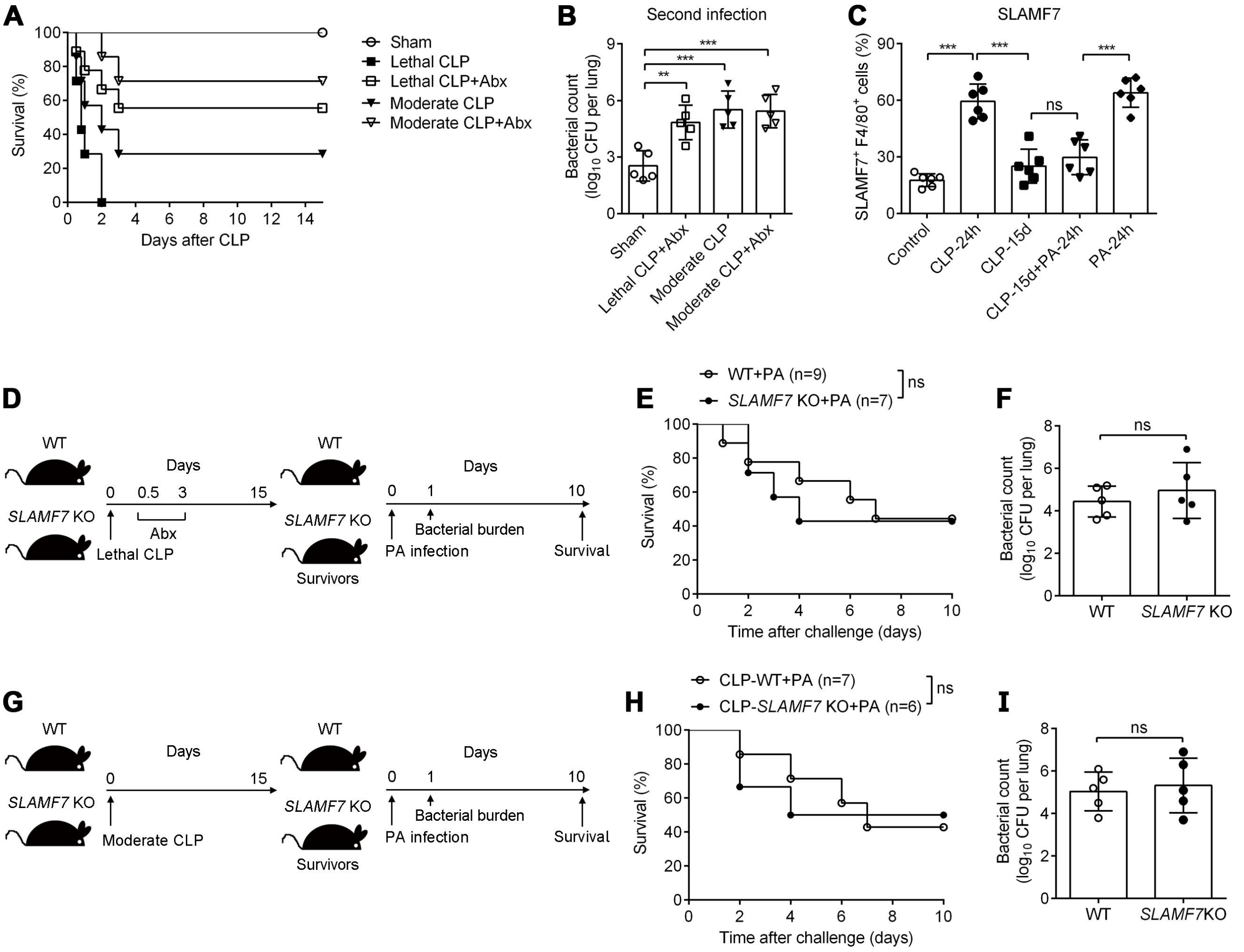

Supplementary Figure 13. SLAMF7 was not involved in sepsis-induced immunosuppression. **A-C**, Lethal and moderate CLP models were respectively established with or without the treatment of antibiotics (Abx) ertapenem sodium. Sepsis-surviving mice were challenged with PA (2x10^7 CFU/kg) 15 days after CLP. (**A**) Survival curves were recorded after CLP challenge (n=7 mice per group). (**B**) After PA infection for 24 hours, the bacterial counts in the lung of sepsis mice were calculated by CFU assay. (**C**) The percentage of SLAMF7$^+$F4/80$^+$ cells in PL was determined by flow cytometry at different time points post CLP as indicated. **D-F**, Lethal CLP surgery with antibiotics was performed in WT and *SLAMF7* KO mice. After 15 days, surviving mice were i.p. infected with PA. (**D**) Schematic of the establishment of immunosuppression sepsis model (lethal CLP with antibiotics). (**E**) Survival rate of mice challenged with PA after CLP. (**F**) Lung bacterial counts in mice challenged with PA. **G-I**, Moderate CLP surgery without antibiotics was conducted in WT and *SLAMF7* KO mice for 15 days, followed by the secondary challenge with PA. (**G**) Schematic of the establishment of immunosuppression sepsis model (moderate CLP without

antibiotics). (**H**) Survival rate was observed. (**I**) Bacterial loads in the lung were determined after PA infection. Data represent the mean ± SEM from at least 3 independent experiments. ns, not significant. $^{**}P< 0.01$, and $^{***}P<0.001$, by one way ANOVA (**B** and **C**), Log-rank test (**E** and **H**) and unpaired Student's t test (**F** and **I**).

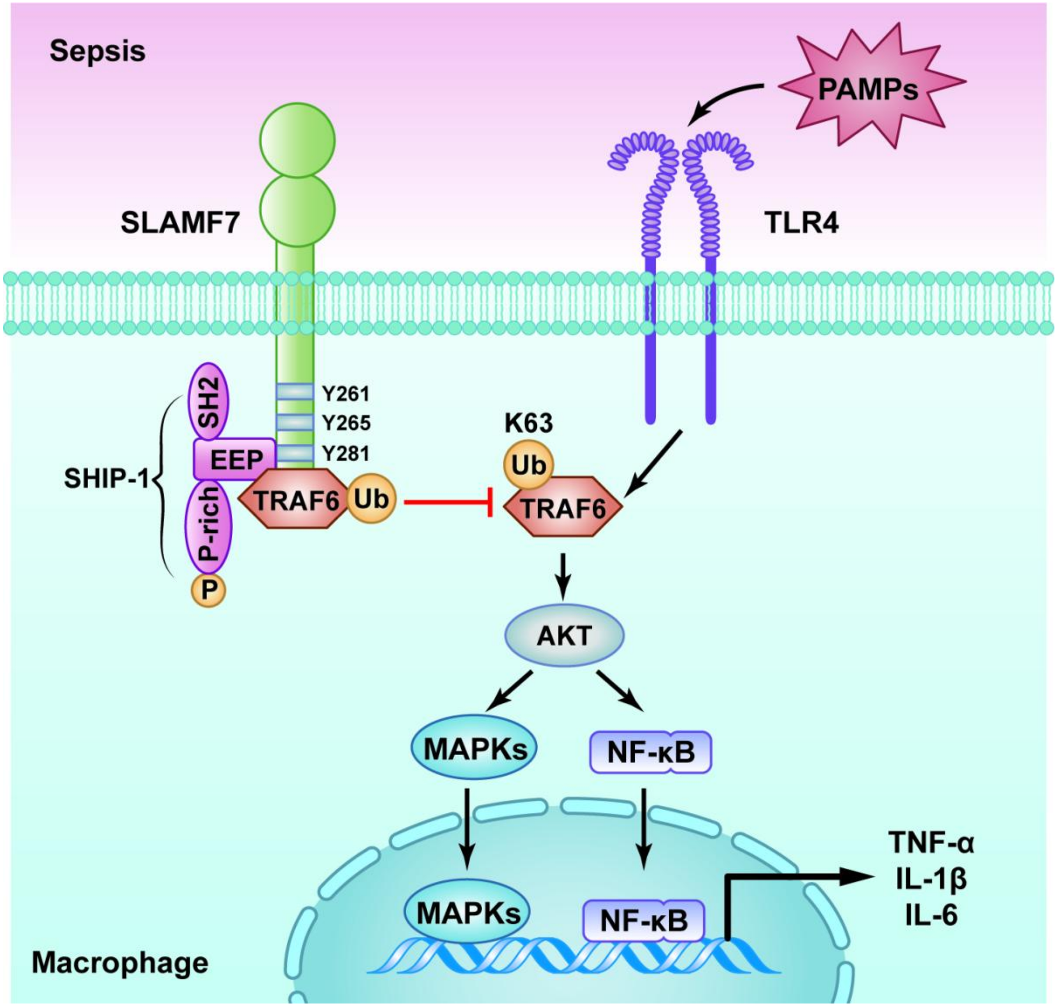

Supplementary Figure 14. Graphical Abstract. During the acute phase of sepsis, TLRs signal (e.g. TLR4) is activated by pathogen associated molecular patterns (PAMPs), resulting in pro-inflammatory responses in macrophage. In this process, the expression of SLAMF7 is up-regulated in monocytes / macrophage. After self-ligand activation, SLAMF7 recruits and induces the phosphorylation of SHIP1 through Y281 site of cytoplasmic domain. Phosphorylated SHIP1 inhibits K63 ubiquitination of TRAF6 through EEP domain, which induced by TLR4 signal. Finally, SLAMF7-SHIP1 signal axis negatively regulates MAPKs and NF-κB signaling pathways, reducing the secretion of pro-inflammatory cytokines including TNF-α, IL-1β and IL-6.

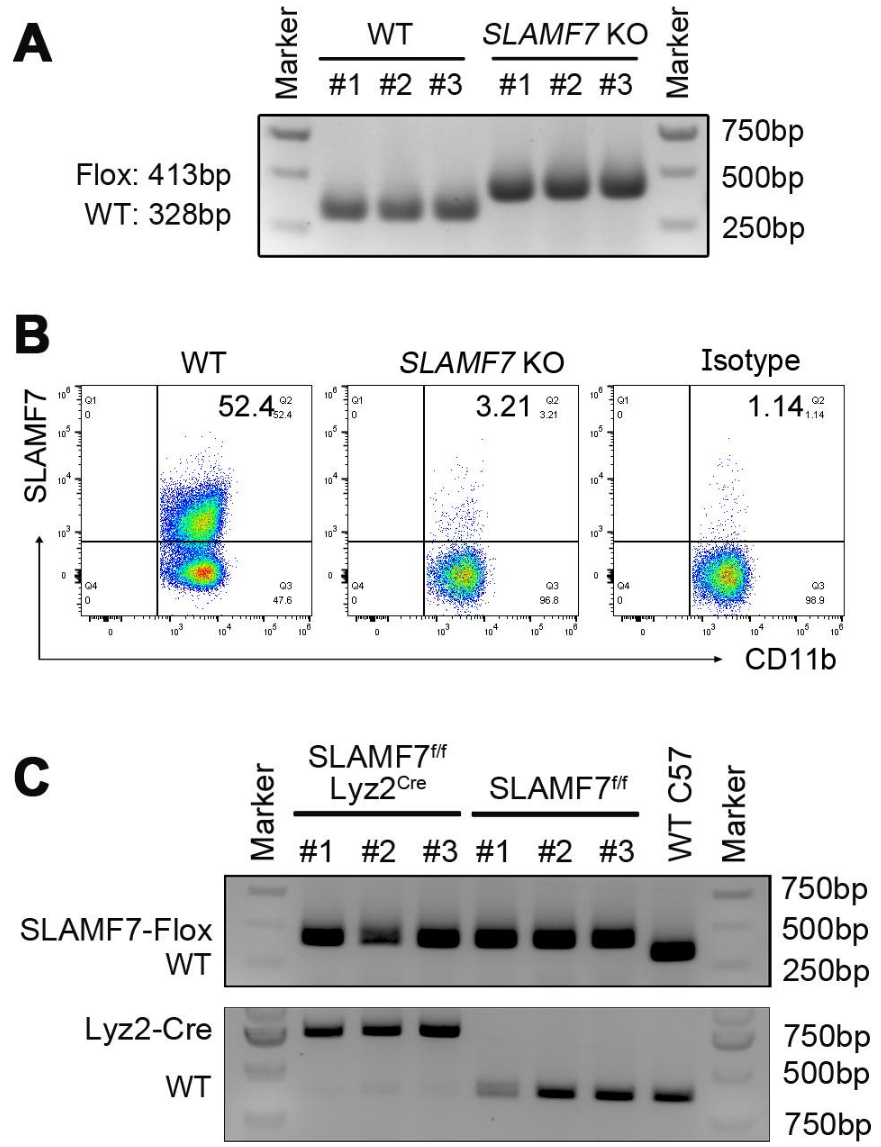

Supplementary Figure 15. Generation of SLAMF7 knockout mice. (A-B) *SLAMF7* KO mice was generated by crispr cas9 system to delete the exon 3/4/5/6 within *Slamf7* gene locus. (**A**) *Slamf7* expression at mRNA level was determined by PCR. (**B**) The protein expression of SLAMF7 in splenic CD11b^{+}cells from WT or *SLAMF7* KO mice was analyzed by flow cytometry. (**C**) Mice with loxP-flanked alleles of *SLAMF7* exon 3/4/5/6 (SLAMF7$^{f/f}$) were crossed with mice expressing Cre recombinase under the control of a Lyz2 promoter (Lyz2-Cre) to achieve myeloid cell-specific deletion of SLAMF7 (SLAMF7$^{f/f}$Lyz2^{Cre}) in mice. PCR analysis of SLAMF7 with loxP-flanked alleles and Lyz2-Cre recombinase from extracted DNA was shown.

Supplemental methods

Bacterial culture

Pseudomonas aeruginosa (PA, ATCC 19660) was grown in *Pseudomonas* isolation agar (Difco, BD). *Escherichia coli* (*E.coli*, ATCC 25922) was grown in LB agar (Difco, BD). Bacterial in logarithmic growth were collected, counted on agar plates and then resuspended in 0.9% NaCl for following infected experiments.

Cell culture and inhibitor reagents

Murine macrophage-like RAW264.7 cells (ATCC TIB-71), 293T (ATCC CRL-11268) and 293FT (ATCC PTA5077) cells were cultured in DMEM supplemented with 10% fetal bovine serum and 100 U/ml penicillin, 100 μg/ml streptomycin at 37°C with an atmosphere of 5% CO2. NF-kB inhibitor (JSH-23), ERK inhibitor (U0126, Sigma), JNK inhibitor (SP600125), p38 MAPK inhibitor (SB203580), AKT inhibitor (Ly294002), TBK1 inhibitor (BX-795), Src inhibitor (PP2), Btk inhibitor (Ibrutinib) and Syk inhibitor (R406) were obtained from Selleck Chemicals (Houston, TX) except were indicated otherwise. TLR1/2 ligands (Pam3Csk4), TLR7/8 ligands (R848) and TLR3 ligands (poly(I:C)) were obtained from InvivoGen (CA, USA).

BMDM and human monocytes isolation

Murine BMDMs were obtained as previously described (1). Briefly cells were maintained in DMEM supplemented with 10% FBS and 10% L929 cell supernatant as conditioned medium providing macrophage colony stimulating factor. BMDMs were obtained as a homogeneous population of adherent cells after 7 days culture. The purity of the differentiation method was > 95% as a routinely confirmed by flow cytometry. Human monocytes were isolated from peripheral venous blood from healthy subjects. Monocytes were isolated from the peripheral blood mononuclear cells (PBMCs) layer according to Ficoll manufacturers (TBD, Tianjin, China). An ammonium chloride-based lysis (BD Biosciences) was performed to remove erythrocytes. PBMCs were then stained in PBS containing 1% BSA with monoclonal antibodies to CD14 (BD Biosciences) for sorting. Purified monocytes were cultured at $1x10^6$ cells/ml overnight before use.

Retrovirus-mediated gene transduction

For making RAW264.7 stable cell lines, the cDNA encoding full-length mouse SLAMF7 (NCBI Gene ID: 75345) was cloned into pSin-EF2-puro-oligo transfer plasmid. For deletion of SLAMF7

gene in RAW264.7, sgRNAs were cloned into pXPR_001 plasmid according to Zhang's lab lentiCRISPR system (2). Retrovirus expressing the appropriate alleles was produced by 293FT cells co-transfected with the packaging system composed of psPAX2 packaging plasmid and pMD2.G envelope plasmid. 48 h after transfection, viral supernatants were collected and filtered and 2 ml of viral supernatant was used to transducer $5x10^6$ RAW264.7 cells in the presence of 8 μg/ml of polybrene (Sigma, St. Louis, MO). 24 h post viral infection, RAW264.7 cells were selected by puromycin (InvivoGen, CA, USA) for 3 days and then were used for the selection of a single clone. Cells against puromycin were subjected for single clone by folds dilution. The positive percentage of SLAMF7 were detected by flow cytometry. The knockout of SLAMF7 was confirmed by immunoblot compared to wild-type RAW264.7 cells.

Reverse transcription and quantitative PCR assay

RNA was extracted from whole cell lysates with TRIzol (Invitrogen) and was reversely transcribed to cDNA with a First Strand cDNA Synthesis Kit (Thermo Fisher Scientific, Waltham, MA). Quantitative real-time PCR was performed in triplicate determinants with a CFX96 RT-PCR Detection System (Bio-Rad). Primary transcripts were measured with primers that amplify either exon-intron junctions or intronic sequences. Threshold cycle numbers were normalized to triplicate samples amplified with primers specific for β-actin. The primer sequences used in this study are listed in the **Supplemental Table 4**.

ELISA assay

Cell culture medium, mouse serum, peritoneal lavage and organ grinding fluid were collected and cytokines production including TNF-α, IL-1β and IL-6 were measured by murine ELISA Kit (R&D System, Minncapolis, MN) according to the manufacturers' instructions.

Colony forming unit (CFU) assay

Peritoneal lavage fluid (PLF) and blood for bacterial culture were collected as mice were euthanized at 18 hours after CLP. PLF and blood were subjected to serial 10-fold dilutions and cultured at 37°C overnight in 5% sheep blood agar (Teknova). CFUs were quantified by manual counting.

Phagocytosis Assay Assessed by Flow Cytometry

Phagocytosis measured by flow cytometry was performed as described previously (3). Adherent macrophages were then co-cultured with Filmtracer Green Biofilm (FTGB, Invitrogen)- labeled PA

or *E.coli* for 1 hour. Following 1 hour bacterial incubation, cells were washed three times utilizing cold PBS and centrifuged to remove the extracellular bacteria. The. The mean fluorescence intensity (MFI) was calculated with flowjo software and used as a measure of phagocytosis.

Confocal microscopy and histological staining

Cells were grown on Glass Bottom Cell Culture Dish were fixed with 4% paraformaldehyde (BBI Life Science, Shanghai, China) followed by membrane permeabilization, blocking and antibody incubation. Nuclei were stained with 4,6-diamidino-2-phenylindole (DAPI, Invitrogen) and mounted with ProLong Gold antifade reagent from Invitrogen and viewed by confocal microscopy (Zeiss, LSM780, German). For histological section staining, mouse lung tissue samples were fixed in 4% PFA for 24 h and embedded in paraffin. Four-μm-thick sections were deparaffinized and stained with hematoxylin and eosin to determine organ damage and leukocyte infiltration. To calculate the lung injury scores from H&E staining, 5 random high-power fields were independently scored in a blinded fashion for each slice. The features of lung injury, including neutrophils in the alveolar space, neutrophils in the interstitial space, hyaline membranes, proteinaceous debris filling the airspaces and alveolar septal thickening, were determined and weighted according to the relevance ascribed to each feature and then were normalized to the number of fields evaluated (4).

Immunoblot analysis and immunoprecipitation assay

Cells samples were lysed in RIPA buffer with protease and phosphatase inhibitor cocktail (Sigma), and then the protein extracts were separated by SDS-PAGE electrophoresis and transferred to a polyvinylidene fluoride or nitrocellulose membranes. The membranes were incubated overnight at 4°C with antibodies against mouse SLAMF7 (Clone AF4628, R&D), human SLAMF7 (Clone E5C4M, #98611, CST), SHIP1 (Clone D1163, #2728, CST), TRAF6 (Clone E2K9D, #67591, CST), AKT (#9272, CST), Ubiquitination (Clone P4D1, #3936T, CST), HA-Tag (Clone C29F4, #3724S, CST), Flag-Tag (Clone DYKDDDK, #2368S, CST), Myc-Tag (#2276, CST), β-actin or the phosphorylation of IKKα/β (Clone 16A6, #2697S, CST), SHIP1 (#3941S, CST), AKT (#2965S, CST), JNK (#4668S, CST), ERK (#4370S, CST) and p-38 (#9215S, CST). Then the membranes were incubated with appropriate HRP-conjugated secondary antibodies (R&D) followed by the visualization with PlusECL (KeyGEN BioTECH). Alternatively, membranes were detected with IRDye 800CW conjugated anti-rabbit IgG or IRDye 680CW conjugated anti-mouse IgG secondary

antibodies (LI-COR Biosciences, Lincoln, NE), and visualized using Odyssey infrared imaging system (LI-COR Biosciences). All antibodies were purchased from Cell Signaling Technology (Beverly, MA, USA) or Abcam if otherwise indicated. For endogenous immunoprecipitation, the protein G plus protein A agarose suspension was purchased from Calbiochem (USA). The anti-Flag M2 affinity gel (#A2220, Sigma) and the anti-HA agarose beads (AT0079, CMCTAG)were used for the other immunoprecipitation assay.

Flow cytometry assay

Single-cell suspensions were counted and collected. For surface staining, cells were stained for human CD11b (Clone ICRF44, Biolegend), CD14 (Clone S18004B, Biolegend), CD56 (Clone 5.1H11, Biolegend), CD3 (Clone UCHT1, Biolegend) and SLAMF7 (Clone 162.1, Biolegend) for the clinical samples, mouse CD11b (Clone M1/70, Biolegend), CD3 (Clone 145-2C, Biolegend), F4/80 (Clone BM8, Biolegend) and SLAMF7 (Clone 4G2, Biolegend) for mouse samples in PBS containing 1% BSA for 20 min on ice in the dark and then washed. Dead cells were excluded using viability dye 506 (#65-0866-18, eBioscience), then washed three times with FACS buffer and fixed in a solution of 1% paraformaldehyde (BBI Life Science, Shanghai, China). In some cases, sing-cell suspensions were stimulated with LPS (L4524, Sigma) in combination with brefeldin A (GolgiPlug, BD Biosciences). After 6 h, cells were surface stained for antibodies then washed twice with FACS buffer stained with fluorochrome-conjugated antibodies against IL-6 (Clone MQ2-13A5, Biolegend), IL-1β (Clone NJTEN3, eBioscience), TNF (Clone MP6-XT22, Biolegend), or their appropriate isotype controls rat IgG1, rat IgG2b for 40min in fixation/permeabilization buffer set (eBioscience, San Diega, CA). Mouse single-cell suspensions were stained with fluorochrome-conjugated antibodies against surface markers. All antibodies were purchased from eBioscience or Biolegend (CA, USA) except where otherwise indicated. All the flow cytometry assays were performed on FACS Canto II (BD, NJ, USA), and data were analyzed by Flowjo software.

Supplemental Table 1. Characteristics and blood parameters of healthy donors and sepsis patients

	Healthy control subjects	Patients with sepsis	*p* value*
Sample size (no.)	81	83	NA
Age (years)	45 (18, 65)	52(15, 75)	NS
Sex (M/F)	(40/41)	(49/34)	NS
Hemoglobin (g/L)	118.26±2.54	90.09±2.72	<0.0001
Platelets ($\times10^9$/ L)	312(181, 493)	246.23 (12,675)	NS
WBC ($\times10^9$/ L)	7.70 (3.7, 11.8)	11.71 (0.2,78.5)	<0.0001
WBC (%)			
Neutrophil (%)	38 (21, 69)	59.9 (6,94)	<0.0001
Monocyte (%)	5.09 (3, 8)	6.66 (0,22)	NS
Lymphocyte (%)	53.74 (21, 72)	31.28 (6,81)	<0.0001
Eosinophil (%)	3.04 (0, 12)	0.72 (0,4)	<0.0001
Basophil (%)	0 .09 (0, 1)	0.33 (0,6)	NS

F, Female; M, male; WBC: White blood cell; NA, not applicable; NS, not significant. Data were shown by mean ± SD or median (25% percentile, 75% percentile). *The level of significance was evaluated by unpaired student *t* test or Mann-Whitney 2-tailed *U* test. *p* value <0.05 was considered statistically significant.

Supplemental Table 2. Clinical characteristics of sepsis patients

Characteristics	No. of Patients [a]
SOFA score, median (range)	8 (4-11)
SASPII score, median (range)	42 (30-54)
Days in hospital, median (range)	38 (8-59)
Days in intensive care unit, median (range)	9 (1-31)
Days of sepsis, median (range) [b]	7 (2-21)
Site of infection	
Intrapelvic abscess	4 (4.8%)
Intravascular catheters	11 (13.3%)
Necrotizing fasciitis	2 (2.4%)
Osteomyelitis	1 (1.2%)
Pneumonia	29 (34.9)
Peritonitis	11 (13.3%)
Retroperitoneal abscess	9 (10.8%)
Urinary tract infection	7 (8.4%)
Comorbidities	
Diabetes	5 (6.0%)
Heart disease	20 (24.1%)
Neurologic	1 (1.2%)
Renal disease	8 (9.6%)
Respiratory	18 (21.7%)
Liver	24 (28.9%)
Others	7 (8.4%)
Organ failure	
Circulatory, vasopressors	17 (20.5%)
Hepatic	15 (18.1%)
Renal	19 (22.9%)
Respiratory	20 (24.1%)
Others	12 (14.5%)
Microbe	
Gram-positive	18 (21.7%)
Gram-negative	44 (53.0%)
Fungal	8 (9.6%)
Others	13 (15.7%)

a Data are expressed as No. of patients unless otherwise indicated.

b Some patients may have been sepsis prior to hospital admission.

Supplemental Table 3. The sequences of genes used in assays of siRNA knockdown.

Target Gene Name	siRNA sequences
SLAMF7-si-001	CCAGGAATCCAGTCAGCAA
SLAMF7-si-002	CCATGAAGCTCAGCCAATT
SLAMF7-si-003	GCAGAGATTTACAGTACAT
SHIP1-si-001	GCAGATGAAGAACAAGCAT
SHIP1-si-002	GCATATCCTGATCAGCATT
SHIP1-si-003	CCAGTGGAATGAAATGCTT
Myd88-si-001	GGAGATGGGCTTCGAGTACTT
Myd88-si-002	GTTAGACCGTGAGGATATACT
Myd88-si-003	GGAGGACTGCCAGAAATACTT

Supplemental Table 4. The primers of genes used in quantitative PCR.

Gene Name	Quantitative Primers（5'-3'）
SLAMF7 (human)	CGGGACCTGCACCTTGATACR-GTTGCTGA<br>TAGGGTTGCTCAC
β-actin (mouse)	GATTACTGCTCTGGCTCCTAGC<br>GACTCATCGTACTCCTGCTTGC
SLAMF7 (mouse)	ACAAGAATGGCACCTGCGTA<br>AGATCCGTGGCAGCATCTTC
SHIP1 (mouse)	CCAGGGCAAGATGAGGGAGA<br>GGACCTCGGTTGGCAATGTA
TNF (mouse)	CACAGAAAGCATGATCCGCGAC<br>TGCCACAAGCAGGAATGAGAAGAG
IL-6 (mouse)	TTCCTCTCTGCAAGAGACTTCCATC<br>GCCTCCGACTTGTGAAGTGGTATAG
IL-1β (mouse)	AGATCCGTGGCAGCATCTTC<br>CGCAGCAGCACATCAACAAGAGC
IL-10 (mouse)	AGATCCGTGGCAGCATCTTC<br>AGATCCGTGGCAGCATCTTC
SAP (mouse)	ATGCAGTGACTGTGTACCACG<br>AGGGACACTCTCGCTGTCT
EAT-2 (mouse)	CTGCCTGACCAAGCGAGAG<br>TCTGAAGATTCGGTAGCTGTAGA

References

1. Zhu S, Chen Y, Lao J, Wu C, Zhan X, Wu Y, et al. Signaling Lymphocytic Activation Molecule Family-7 Alleviates Corneal Inflammation by Promoting M2 Polarization. *J Infect Dis.* 2021;223(5):854-65.

2. Ran FA, Hsu PD, Wright J, Agarwala V, Scott DA, and Zhang F. Genome engineering using the CRISPR-Cas9 system. *Nat Protoc.* 2013;8(11):2281-308.

3. Wu Y, Li D, Wang Y, Liu X, Zhang Y, Qu W, et al. Beta-Defensin 2 and 3 Promote Bacterial Clearance of Pseudomonas aeruginosa by Inhibiting Macrophage Autophagy through Downregulation of Early Growth Response Gene-1 and c-FOS. *Front Immunol.* 2018;9:211.

4. Matute-Bello G, Downey G, Moore BB, Groshong SD, Matthay MA, Slutsky AS, et al. An official American Thoracic Society workshop report: features and measurements of experimental acute lung injury in animals. Am J Respir Cell Mol Biol. 2011;44(5):725-38.
